# Mechanistic insights into lactate-driven pathogenesis in diabetic kidney disease

**DOI:** 10.3389/fphar.2026.1950569

**Published:** 2026-10-22

**Authors:** Shihao He, Longyan Yang, Ruili Yin

**Affiliations:** 1 Beijing Luhe Hospital, Capital Medical University, Beijing, China; 2 Center for Endocrine Metabolism and Immune Diseases, Beijing Luhe Hospital, Capital Medical University, Beijing, China; 3 Beijing Key Laboratory of Artificial Intelligence and Cell-based Medical Engineering for Interdisciplinary Innovation and Clinical Translation, Beijing, China; 4 Laboratory for Clinical Medicine, Capital Medical University, Beijing, China

**Keywords:** diabetic kidney disease, lactate, lactylation, metabolic reprogramming, renal fibrosis

## Abstract

Diabetic kidney disease (DKD) remains the leading cause of end-stage renal disease worldwide, yet current therapeutic approaches fail to fully halt its progression. Emerging evidence identifies lactate not merely as a glycolytic byproduct, but as a critical signaling molecule that drives DKD pathogenesis via lysine lactylation. This review delineates the mechanistic cascade from upstream metabolic dysregulation to epigenetic reprogramming and renal injury. We first describe how the hyperglycemic and hypoxic microenvironment stabilizes hypoxia-inducible factor-1α (HIF-1α), initiating a positive feedback loop that amplifies glycolytic flux and lactate accumulation. Then, we discuss the dynamic regulation of lactylation by “writer” enzymes and “eraser” enzymes. Furthermore, we analyze how lactate-induced lactylation may contribute to metabolic and immune-inflammatory remodeling across key renal cells: promoting podocyte epithelial-mesenchymal transition (EMT) and autophagy suppression; driving tubular fibrosis and ferroptosis via specific lactylation axes; and activating glomerular endothelial NOD-like receptor family pyrin domain-containing 3 (NLRP3) inflammasomes. Beyond lactylation, we address other lactate-mediated mechanisms, including GPR81 signaling and intracellular acidification. Finally, we evaluate therapeutic strategies targeting this pathway-such as SGLT2 inhibitors and traditional Chinese medicine-and discuss the challenges in clinical translation. By integrating the latest mechanistic advances, this review discusses lactate-induced lactylation as a potential hub that may connect metabolic, epigenetic, and immune injury, while acknowledging that renal inflammation in DKD arises from diverse upstream pathways, providing a roadmap for the development of next-generation biomarkers and targeted therapies.

## Highlights

- Lactate as a signaling nexus: Lactate functions as a critical signaling molecule that drives diabetic kidney disease (DKD) pathogenesis through metabolic dysregulation, epigenetic remodeling, and immune-inflammatory signaling.
- Self-amplifying injury loop: Lysine lactylation establishes a self-amplifying feedforward loop, notably via HIF-1α stabilization, which perpetuates progressive renal fibrosis and failure across multiple resident kidney cells.
- Novel therapeutic potential: Targeting the lactate-lactylation axis—including writers, erasers, and modulators like SGLT2 inhibitors—offers a promising avenue for novel diagnostics and targeted renoprotective interventions.

## 1 Introduction

Diabetic kidney disease (DKD) is a common microvascular complication in patients with diabetes and a leading cause of end-stage renal disease (ESRD) worldwide (Bloomgarden, 2005). It is typically characterized by persistent albuminuria and a progressive decline in the glomerular filtration rate (GFR), ultimately progressing to ESRD. Epidemiological data indicate that DKD affects approximately 25%–40% of the diabetic population (Alicic et al., 2017; Jung and Yoo, 2022). Notably, its prevalence has not declined over the past 3 decades, posing a severe and growing public health challenge to the expanding patient population (Jung and Yoo, 2022). Evidence suggests that in patients with diabetes and chronic kidney disease (CKD), a higher urinary lactate-to-creatinine ratio is strongly associated with a decline in the glomerular filtration rate, indicating a potential key role for lactate in the progression of diabetic kidney disease (Azushima et al., 2023; Darshi et al., 2024). In db/db induced DKD mice, sodium-glucose cotransporter 2 inhibitors (SGLT2i) alter kidney immune status: they raise anti-inflammatory macrophages and reduce harmful tubular–immune cell crosstalk to improve renal function (Bai et al., 2024). Cui et al. reported that dapagliflozin may attenuate podocyte injury and proteinuria in DKD, at least in part by suppressing lactate generation and H4K12 lactylation (H4K12la) in podocytes (Cui et al., 2026). Collectively, these findings raise the intriguing possibility that lactate, along with its derived lysine lactylation, may serve as a metabolic link between SGLT2i-induced immune remodeling and the attenuation of DKD progression, highlighting the potential centrality of lactate-mediated metabolic-immune crosstalk in diabetic kidney disease.

Accumulating evidence suggests that metabolic disorders are profoundly shaped by systemic metabolic reprogramming initiated in the gut, where dysbiosis drives host metabolic alterations via microbial-derived metabolites (Wang et al., 2026). Notably, lactate is a key end-product of gut microbial glucose metabolism, and its overproduction contributes to systemic low-grade inflammation. This framework provides a theoretical basis for exploring whether gut-derived metabolites affect lactate accumulation and lactylation levels in DKD.

DKD arises from the interplay of multiple mechanisms, including metabolic disturbances, mitochondrial damage, and immune-inflammatory responses. Metabolic reprogramming, also known as the Warburg effect, was first described by Otto Warburg in the 1920s, referring to the phenomenon where tumor cells preferentially utilize glycolysis for ATP synthesis even under aerobic conditions (Warburg et al., 1927). With advancing research technologies, accumulating evidence has demonstrated that metabolic reprogramming also plays a crucial role in the development of DKD. The hyperglycemic and hypoxic microenvironment in DKD drives a shift in renal energy metabolism from reliance on mitochondrial oxidative phosphorylation (OXPHOS) and fatty acid oxidation (FAO) toward aerobic glycolysis. This metabolic shift leads to renal lactate accumulation, mitochondrial dysfunction, and dysregulated immune inflammation (Wang et al., 2022). Notably, studies have revealed that lactate is associated with the progression of DKD by inducing lactylation at the lysine 206 (K206) site of tripartite motif-containing 65 (TRIM65), which may activate glycolysis and triggers ferroptosis in renal tubular epithelial cells (Yang G. et al., 2025). In a study on lupus nephritis, lactate was found to induce lysine lactylation at the K40 site of pre-B-cell leukemia homeobox transcription factor 1 (PBX1), thereby enhancing the interaction between PBX1 and TRIM21. This interaction promotes ubiquitination at the K29 and K63 sites, leading to PBX1 degradation, downregulation of the cell cycle inhibitor p27 transcription, and ultimately mesangial cell hyperproliferation (Liu et al., 2025). These findings collectively demonstrate that lactate not only serves as the end product of aerobic glycolysis but also functions as a key signaling molecule involved in important epigenetic regulation.

Therefore, this review will provide a comprehensive and systematic exploration of the role of lactate in diabetic kidney disease, spanning from its accumulation and lactylation processes to the consequent renal damage. It will further discuss current therapeutic advances and potential treatment targets in DKD, aiming to offer valuable insights for the global prevention and management of this condition.

## 2 Review methods

This study was designed as a structured narrative review following the SANRA (Scale for the Assessment of Narrative Review Articles) principles to ensure methodological rigor, transparency, and reproducibility. Because this article aims to provide a comprehensive mechanistic synthesis rather than a pooled statistical analysis, a meta-analysis was not conducted. A systematic search was conducted across electronic databases, including PubMed, Web of Science (Core Collection), and the China Biology Medicine disc (CBM), from their inception to 25 August 2026. The search was limited to peer-reviewed articles published in English. The search algorithm utilized a combination of Medical Subject Headings (MeSH) and free-text terms in the “Title/Abstract” fields, combined via Boolean operators (AND, OR): Disease and Organ Terms: “Diabetic Kidney Disease”, “Diabetic Nephropathy”, “Renal”, “Kidney”, “Diabetic Complications”; Metabolic and Modification Terms: “Lactate”, “Lactic Acid”, “Lactylation”, “Lysine Lactylation”, “Histone Lactylation”, “Metabolic Reprogramming”, “Warburg Effect”; Enzymatic and Pathway Regulators: “p300”, “EP300”, “Histone Deacetylases” (“HDAC”), “Sirtuins” (“SIRT”); Pathophysiological Outcomes: “Fibrosis”, “Inflammation”, “Oxidative Stress”, “Therapeutics”.

Studies were included if they met the following criteria: (1) Original research articles (*in vivo* animal models, *in vitro* cell experiments, human clinical or epidemiological studies) and systematic reviews/meta-analyses; (2) Studies investigating lactate production, lactate signaling or lactate-induced lactylation in kidney disease, diabetes, or metabolic disorders; (3) Studies establishing the enzymatic basis of lactylation (writer/eraser identification) in any disease model, provided the mechanism is relevant to renal pathophysiology. In addition, the reference lists of retrieved original articles and relevant systematic reviews were manually screened (“snowballing”) to identify pertinent studies.

All retrieved citations were imported into Zotero for deduplication and management. Literature screening was conducted in two sequential stages: (1) Initial screening: Two independent reviewers screened all identified records by title and abstract. (2) Full-text evaluation: Potentially relevant articles were retrieved for full-text evaluation. Discrepancies regarding study eligibility were resolved by consensus or through adjudication by a third senior reviewer.

Inclusion criteria: (1) Primary DKD Evidence: *In vivo* animal models, *in vitro* cell culture systems, and human clinical or epidemiological studies investigating lactate accumulation, lactate transport, or lactylation modifications in the context of diabetic kidney disease and metabolic disorders. (2) Fundamental Mechanistic Studies (Non-DKD Evidence): Landmark and mechanistic studies in non-DKD fields (e.g., oncology, acute kidney injury [AKI], sepsis, ischemic stroke). (3) Secondary Literature: High-quality reviews and meta-analyses offering validated pathway frameworks or therapeutic benchmarks.

Exclusion criteria: (1) Studies unrelated to lactate metabolism, lactylation modifications, or renal/metabolic pathophysiology. (2) Conference abstracts, case reports, letters to the editor, editorials, and preprints lacking primary experimental data or detailed methodologies. (3) Duplicate publications or studies with unavailable full texts.

## 3 Lactate metabolism remodeling in DKD

In DKD, the hyperglycemic and hypoxic microenvironment drives the initiation of metabolic reprogramming. Subsequently, the enhanced activity of various rate-limiting enzymes and signaling molecules progressively increases the glycolytic flux in renal cells, leading to lactate accumulation. Excessive lactate buildup may drive the lactylation of multiple signaling molecules and rate-limiting enzymes, which in turn activate downstream signaling pathways. This cascade may result in progressive metabolic abnormalities and pathological alterations. The following sections will focus on the accumulation and functional role of lactate, elucidating how the hyperglycemic milieu ultimately leads to pathological changes in the kidney.

### 3.1 Glycolytic reprogramming and lactate accumulation

#### 3.1.1 The hyperglycemic and hypoxic environment drives the stabilization and activation of HIF-1α

Hypoxia-inducible factor-1 (HIF-1) is a key mediator of the physiological response to hypoxia. HIF-1 is a heterodimeric protein composed of two subunits: HIF-1α and HIF-1β (Iyer et al., 1998). Under normoxic conditions, the HIF-1α protein is continuously synthesized; however, in the presence of oxygen, it undergoes post-translational hydroxylation at proline residues 402 and 564 by oxygen-dependent prolyl hydroxylases (PHDs). The hydroxylated proline residues of HIF-1α are then recognized by the E3 ubiquitin ligase adaptor protein von Hippel-Lindau (VHL), leading to polyubiquitination of HIF-1α and its subsequent degradation by the 26S proteasome (Li et al., 2025). Under pathological conditions, a hypoxic environment can directly stabilize HIF-1α by inhibiting PHD activity, subsequently upregulating the expression of key glycolytic genes, driving metabolic reprogramming, and leading to lactate accumulation (Tiwari et al., 2024). Notably, DKD is characterized not by isolated HIF-1α activation, but rather by a shift in the HIF-1α/HIF-2α activity balance—manifested as aberrant HIF-1α upregulation coupled with concurrent HIF-2α suppression—a reversal that promotes pro-inflammatory and pro-fibrotic pathways in both glomerular and tubular compartments (Stanigut et al., 2022). This isoform-specific divergence is further shaped by the renal oxygen tension gradient: cortical tissue maintains a relatively oxygenated environment, whereas the medullary region operates under physiological hypoxia. Diabetes exacerbates this heterogeneity, and SGLT2i redistributes hypoxic zones by altering tubular oxygen consumption (Hesp et al., 2020). Temporally, HIF-1α and HIF-2α exhibit distinct kinetics. HIF-1α is induced during the initial adaptive phase to hypoxia, whereas HIF-2α expression emerges only after prolonged hypoxic exposure, suggesting that the HIF-1α/HIF-2α ratio evolves dynamically across DKD stages (Stanigut et al., 2022).

Mitochondrial damage is a key factor triggering aerobic glycolysis. Under hyperglycemic conditions, the overloaded mitochondrial electron transport chain reduces electron transfer efficiency in oxidative phosphorylation. Damage to the electron transport chain leads to excessive production of reactive oxygen species (ROS), which stabilize HIF-1α and upregulate the expression of glycolytic genes. Excess ROS also attacks proteins of the mitochondrial respiratory chain complexes, further impairing oxidative phosphorylation (Yao et al., 2022). However, the HIF-1α–ROS relationship is not unidirectional. In proximal tubular cells, HIF-1α exerts protective feedback by upregulating heme oxygenase-1 (HO-1), which preserves mitochondrial dynamics through increased mitofusin-1/2 (Mfn1/2) expression and suppressed dynamin-related protein 1 (Drp1) activation, thereby limiting ROS overproduction and apoptosis (Jiang et al., 2020). Complementarily, HIF-1α promotes mitophagy in renal tubular cells exposed to high glucose, clearing damaged mitochondria and further attenuating ROS accumulation and epithelial-mesenchymal transition (Stanigut et al., 2022). These findings reveal a cell-type- and context-dependent duality: while HIF-1α is widely expressed across tubular segments and can limit mitochondrial injury in proximal tubules, its role in podocytes is predominantly maladaptive. Chronic HIF-1α accumulation drives foot-process effacement, slit-diaphragm protein loss, and epithelial-mesenchymal transition (Stanigut et al., 2022).

Concurrently, prolonged exposure of renal cells to hyperglycemia enhances glycosylation reactions and increases the accumulation of advanced glycation end products (AGEs), aggravating renal injury. This process promotes the synthesis of AGEs, which activate the receptor for AGEs (RAGE). The AGE-RAGE axis stimulates the generation of oxygen free radicals, further stabilizing HIF-1α. Studies have shown that the AGE-RAGE axis can also activate signaling pathways such as NF-κB, mitogen-activated protein kinase/extracellular signal-regulated kinase (MAPK/ERK), and phosphoinositide 3-kinase/protein kinase B (PI3K/Akt), ultimately contributing to glomerular damage, mesangial expansion, and proteinuria (Wu et al., 2023; Al-Masri et al., 2026). These pathways are well-established mediators of inflammation and fibrosis in DKD. Notably, metabolic reprogramming toward aerobic glycolysis and subsequent lactate accumulation is another hallmark of DKD. Although phosphatase and tensin homolog (PTEN), the primary endogenous antagonist of PI3K/Akt signaling, has been reported to be dysregulated in hypertensive kidney disease, leading to sustained PI3K/Akt hyperactivation, a glycolytic shift, and excessive lactate accumulation (An et al., 2022), direct evidence linking the AGE-RAGE axis to lactate production in the context of DKD remains limited. Therefore, while both AGE-RAGE signaling and lactate accumulation contribute to DKD pathogenesis, their potential interplay requires further investigation.

#### 3.1.2 HIF-1α drives the transcriptional activation of glycolytic processes

Overactivation of glycolytic genes can lead to excessive lactate accumulation, in which the PKM2-HIF-1α feedback loop plays a critical role. In a hyperglycemic environment, stabilization and activation of HIF-1α upregulate the expression of various glycolytic genes, including glucose transporter 1 (GLUT1), lactate dehydrogenase A (LDHA), and pyruvate kinase M2 (PKM2) (Li Z. et al., 2026). Importantly, PKM2 functions not only as a metabolic enzyme but also as a key signaling regulator. PKM2 can shift from a highly glycolytically active tetrameric form to a transcriptionally active dimeric form. The dimeric PKM2 translocates into the nucleus, where it interacts with HIF-1α and enhances the transcriptional activity of glycolytic genes such as GLUT1 and LDHA (Gong et al., 2025). Studies indicate that elevated HIF-1α levels can alter the expression profile of 6-phosphofructo-2-kinase/fructose-2,6-biphosphatase (PFKFB) genes, promoting the conversion of phosphofructokinase-2 (α-PFK2) to more active isozyme forms, thereby increasing glycolytic flux and ultimately resulting in excessive lactate accumulation (Rodríguez-Prados et al., 2010; Ruiz-García et al., 2011). Additionally, research using myeloid-specific 6-phosphofructo-2-kinase/fructose-2,6-biphosphatase 3 (PFKFB3) knockout mice demonstrated that glycolytic metabolites activated by PFKFB3 can stabilize HIF-1α, upregulate the expression of IL-1β and TNF-α, and ultimately contribute to renal fibrosis (Yang et al., 2023). The mechanisms leading to lactate accumulation are summarized in Figure 1.

**FIGURE 1:**
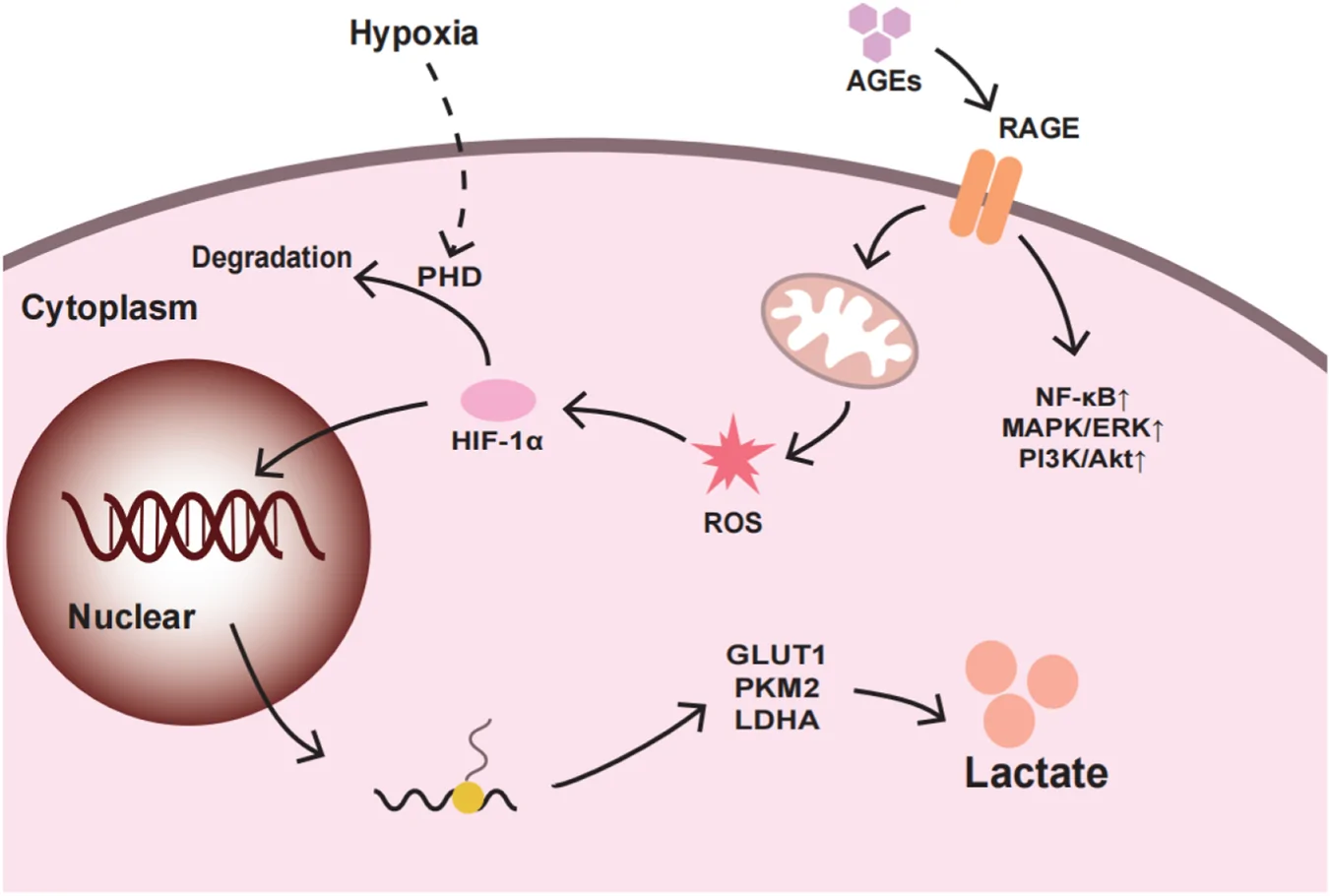
Schematic overview of metabolic reprogramming and lactate action in diabetic kidney disease Hypoxia and accumulating AGEs cooperatively drive a feed-forward pathogenic cycle. Hypoxia directly inhibits the enzymatic activity of PHD, while AGEs engage their cognate receptor RAGE. This engagement amplifies ROS generation and activates downstream NF-κB, MAPK/ERK, and PI3K/Akt signaling cascades, precipitating renal inflammation. Critically, ROS derived from both the hypoxic and AGE-RAGE axes exert synergistic effects to suppress PHD function, thereby stabilizing the structural integrity of HIF-1α and precluding its proteasomal degradation. Stabilized HIF-1α translocates to the nucleus, where it transcriptionally upregulates key glycolytic enzymes (such as PKM2 and LDHA) and the glucose transporter GLUT1. This glycolytic reprogramming drives excessive lactate production, which further exacerbates ROS accumulation and inflammatory signaling, establishing a self-sustaining vicious cycle. The resultant lactate surplus provides the obligate substrate for both lactylation-dependent and -independent pathogenic mechanisms detailed in subsequent figures. Abbreviations: DKD, diabetic kidney disease; HIF-1α, hypoxia-inducible factor-1α; GLUT1, glucose transporter 1; PKM2, pyruvate kinase M2; LDHA, lactate dehydrogenase A; Kla, lysine lactylation; HDAC1-3, histone deacetylases 1–3; ROS, reactive oxygen species; AGEs, advanced glycation end-products; PHD, prolyl hydroxylases.

### 3.2 Lactate transport and intercellular lactate shuttle

Renal lactate handling is spatially gated by the monocarboxylate transporter (MCT) family of proton-coupled transporters. In mouse kidney, Becker et al. mapped their segment-specific distribution: MCT1 resides on the basolateral membrane of proximal tubular S1–S2 segments, MCT2 and MCT7 are expressed in the thick ascending limb and distal convoluted tubule, while apical sodium-coupled monocarboxylate transporters 1/2 (SMCT1/2) mediate luminal lactate reabsorption (Becker et al., 2010; Halestrap, 2013). This architecture defines the baseline framework for transcellular lactate flux.

Notably, in lupus nephritis models, the MCT-partner protein CD44 is scarcely detectable in healthy tubular epithelia yet is strongly induced on the basolateral membrane of injured tubules (Benz et al., 1996). Its pathological upregulation is poised to potentiate MCT-mediated lactate uptake, expanding the intracellular lactate reservoir that fuels lactylation.

Under glycolytic stress, the low-affinity/high-capacity lactate exporter MCT4 (encoded by the Slc16a3 gene) is induced. In postischemic mouse kidneys, concurrent endothelial inactivation of PHD1–3 drives MCT4 upregulation, exacerbating the maladaptive AKI-to-CKD transition; conversely, pharmacological inhibition of MCT4 with syrosingopine restores adaptive repair by suppressing endothelial inflammation, positioning MCT4 as a critical node governing paracrine lactate shuttling between renal endothelium and parenchyma (Tiwari et al., 2024; Dong et al., 2026).

Collectively, the MCT/CD44 axis governs substrate availability for lactylation: basal tubular lactate tone is gated by MCT1/SMCT-mediated reabsorption, whereas stress-inducible MCT4 export directs paracrine lactate flux to fibrogenic renal cell populations. Notably, the functional architecture of this cascade has been exclusively delineated in non-diabetic kidney injury contexts to date; direct mechanistic validation in DKD-specific preclinical systems thus remains a high-priority unmet need in the field.

## 4 Biological functions of lactate in DKD

### 4.1 Lactate receptor-mediated effects of lactate in DKD

As discussed above, hyperglycemia and hypoxia drive metabolic reprogramming via HIF-1α-dependent upregulation of GLUT1, LDHA, HK2, and PFKFB3, leading to excessive lactate production. In diabetic kidney disease (DKD), lactate itself can exert pathogenic effects through multiple non-lactylation mechanisms. As an endogenous ligand for the G protein-coupled receptor (GPR81), also known as Hydrocarboxylic acid receptor 1 (HCAR1), lactate activates Gi protein-coupled signaling, inhibits adenylyl cyclase activity, and reduces intracellular cAMP levels (Manoharan et al., 2021). GPR81 engagement elicits context-dependent outcomes. In LPS-stimulated macrophages, lactate-GPR81 signaling sequentially activates AMP-activated protein kinase (AMPK) and large tumor suppressor kinase (LATS), promoting phosphorylation and inactivation of Yes-associated protein (YAP). This disrupts the YAP-NF-κB p65 interaction, impairs their co-nuclear translocation, and attenuates NF-κB transcriptional activity, representing an anti-inflammatory axis (Liu et al., 2009; Yang et al., 2020). Conversely, in the renal microenvironment-particularly under ischemic and diabetic conditions-GPR81 activation manifests predominantly injurious effects. In a model of ischemic kidney injury, activation of GPR81 in renal vascular smooth muscle cells and pericytes promotes endothelin-1 (ET-1) release, which acts on the ETA receptor to elevate blood pressure and reduce renal blood flow and glomerular filtration rate (Jones et al., 2020). Genetic ablation of GPR81 attenuates renal ischemia-reperfusion injury, suggesting that the GPR81/ET-1 axis may represent a potential therapeutic target for ischemic nephropathy and may also contribute to non-lactylation-mediated injury in DKD (Jones et al., 2020).

Beyond receptor-mediated signaling, emerging evidence indicates that lactate directly promotes inflammation through intracellular acidification and non-enzymatic cytokine cleavage. Lin et al. demonstrated that in macrophages stimulated with NOD-like receptor family pyrin domain-containing 3 (NLRP3) activators, glycolysis-derived lactate accumulates intracellularly, leading to rapid cytosolic acidification. This acidification promotes NLRP3 inflammasome assembly via two parallel pathways: mitochondrial ROS generation and PKR phosphorylation, the latter of which facilitates PKR–NLRP3 interaction (Lin et al., 2026). Notably, lactate itself can directly cleave pro-IL-1β and pro-IL-18 at Asp116-the canonical caspase-1 cleavage site-through a carboxyl group-dependent, non-enzymatic mechanism, thereby generating mature cytokines independently of inflammasome activation (Shahzad et al., 2022; Gouchoe et al., 2024). Systemic lactate administration during murine sepsis drove NLRP3-dependent inflammation, elevating IL-1β and recruiting neutrophils, which culminated in hypothermia and increased mortality (Lin et al., 2026). These lactate receptor-mediated effects of lactate are summarized in Figure 2.

**FIGURE 2:**
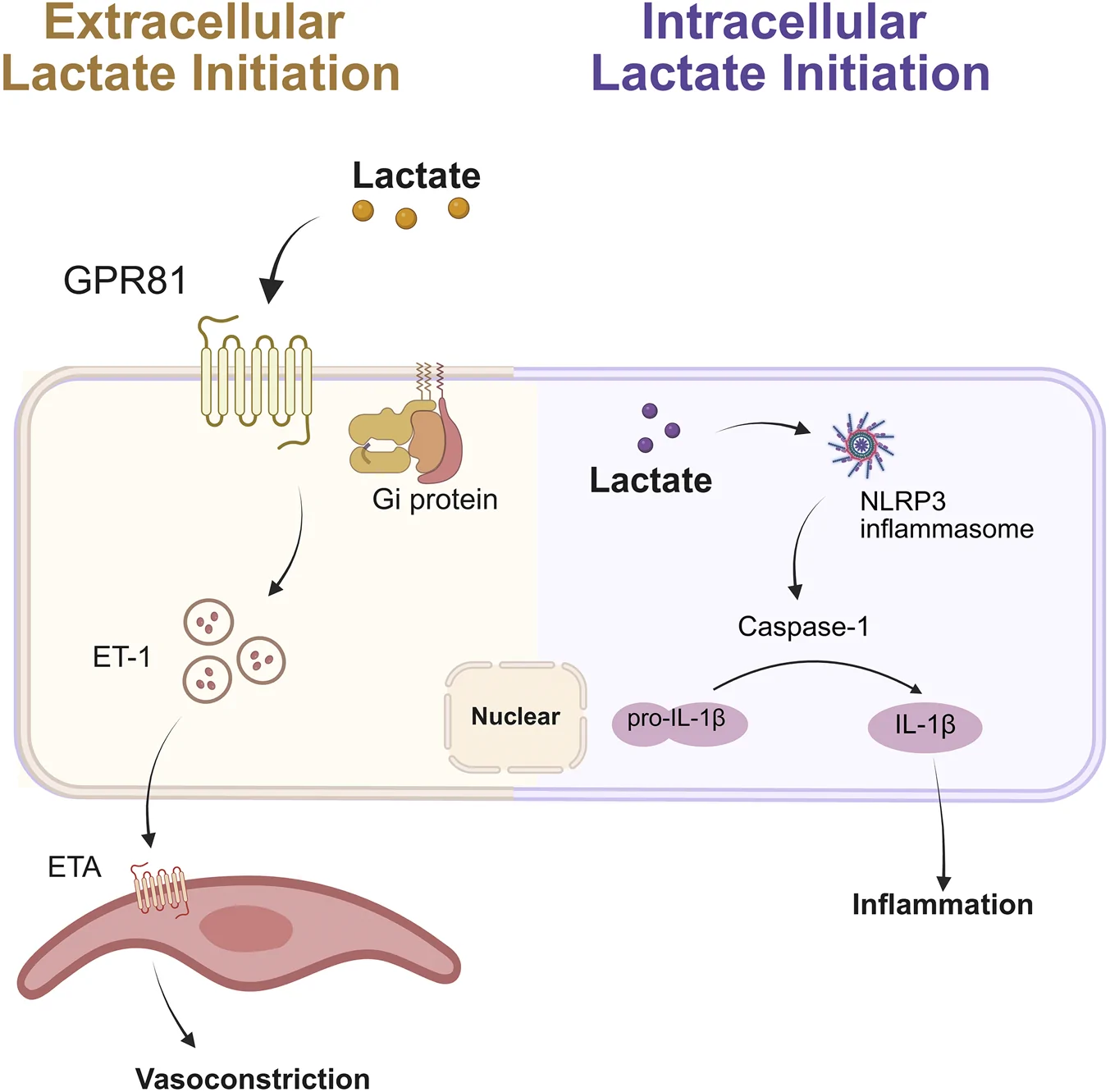
Lactate receptor-mediated effects of lactate in DKD. Lactate drives renal injury through two parallel, receptor-independent and -dependent routes. Left panel: Extracellular lactate activates GPR81 on vascular smooth muscle cells and pericytes, triggering ET-1 release to induce vasoconstriction, reduce RBF and lower GFR. Right panel: Intracellular lactate accumulation causes cytosolic acidification, which promotes NLRP3 inflammasome activation and enables unique carboxyl-dependent non-enzymatic cleavage of pro-IL-1β, generating mature IL-1β to propagate sterile inflammation. These pathways operate alongside lactylation-dependent mechanisms (detailed in subsequent figures) to sustain DKD progression. Abbreviations: DKD, diabetic kidney disease; ET-1, endothelin-1; GPR81, G protein-coupled receptor 81; GFR, glomerular filtration rate; IL-1β, interleukin-1 beta; NLRP3, NOD-like receptor thermal protein domain associated protein 3; RBF, renal blood flow.

### 4.2 Epigenetic regulation of lactate-induced lysine lactylation: the “writer–eraser” system

Lactate-induced lysine lactylation may serve as a bridge connecting metabolic regulation and epigenetic control. It can modulate chromatin activity and alter transcriptional states, thereby regulating renal immune-inflammatory responses and progressively linking metabolic reprogramming to disease progression. This process is regulated by enzymes analogous to “writers” and “erasers” in other acylation systems, though the assignment of specific enzymes to these roles remains context-dependent and continues to evolve.

Intracellular lactate can be converted to lactyl-CoA, which serves as a high-energy donor for the covalent modification of lysine residues, catalyzed by histone acetyltransferases (HATs). Among them, p300/CREB-binding protein (CBP) have been shown to utilize lactyl-CoA as a substrate to catalyze histone lysine lactylation in several cellular contexts (Wei et al., 2025; Li Z. et al., 2026). In a sepsis-induced acute kidney injury (AKI) model, elevated lactate levels enter macrophages via monocarboxylate transporters (MCTs) and mediated by p300/CBP, promote lactylation of high-mobility group box 1 (HMGB1) (Yang et al., 2022). This modification facilitates HMGB1 release from macrophages via exosomes, disrupting vascular endothelial integrity and increasing vascular permeability (Yang et al., 2022). Beyond this lactylation event, a broader epigenetic reprogramming involving the M2 macrophage transcription factor signal transducer and activator of transcription 6 (STAT6) and the histone demethylase Jumonji domain containing-3 (JMJD3) critically remodels the epigenetic landscape during renal immune responses. In chronic kidney disease models, STAT6 activation in renal interstitial cells drives M2 polarization and myeloid fibroblast activation, as demonstrated by both pharmacological inhibition and genetic deficiency studies (Jiao et al., 2021a; Jiao et al., 2021b). JMJD3 functions as an epigenetic executor that removes the repressive H3K27 dimethylation mark at the IFN regulatory factor 4 (IRF4) locus to promote M2 programming and renal fibrosis, representing a parallel epigenetic mechanism to the STAT6-dependent transcriptional program (An et al., 2023). Whether the lactate-lactylation axis intersects with this cascade in the context of diabetic kidney disease remains to be further investigated. In the context of ischemic stroke, experimental evidence has further demonstrated that p300-dependent H3K9 lactylation is locally enriched at the Nrf2 promoter region, driving an anti-inflammatory M2 program in microglia and thereby attenuating neuronal injury. Pharmacological inhibition with C646 or genetic interference abrogates stimulus-induced H3K9la and its downstream transcription, establishing p300 as the catalytic writer in lactate-induced lactylation (Li B. et al., 2026). Studies have shown that knockdown of the p300 gene significantly reduces H4K12la levels, confirming the catalytic role of the p300 protein in lactylation (Deng et al., 2025). However, it should be noted that these validations derive from non-DKD models, and whether p300 serves as the dominant lactyltransferase in specific renal cell types under diabetic conditions awaits direct confirmation.

Beyond the lactyl-CoA-dependent pathway, alanyl-tRNA synthetases (AARSs) can catalyze histone lactylation in a lactyl-CoA–independent manner. Research indicates that AARSs mediate lactylation at histone H3K18 and at K193 of the transcription factor STAT1 in DKD, ultimately promoting lipid peroxidation and ferroptosis (Hong et al., 2026; Zhou et al., 2026). This constitutes the most direct validation of a lactylation writer in DKD to date, though the full spectrum of AARSs substrates in renal cells remains to be systematically defined.

Histone deacetylases (HDACs) have been demonstrated to possess histone delactylation activity, functioning as key “eraser” enzymes in the process of lactate-induced lactylation within the nucleus (Li Z. et al., 2026). Class I HDAC1–3 have been systematically evaluated *in vitro* and all exhibit significant and robust histone delactylation activity, with HDAC3 showing the strongest activity. Quantitative analysis of HDAC3-mediated histone delactylation *in vitro* revealed that, across two replicate experiments, 39 lysine-lactylated peptides were identified (Moreno-Yruela et al., 2022; Fan et al., 2024; Yang Z. et al., 2025). Among these, 31 peptides showed a reduction of over 90% in lactylation levels upon HDAC3 treatment, highlighting its potent delactylating activity (Moreno-Yruela et al., 2022). Functional studies in pancreatic ductal adenocarcinoma cells demonstrate context-dependent substrate selectivity: HDAC2 overexpression, but not HDAC1 or HDAC3, preferentially suppressed both global protein lactylation and histone H3K18 lactylation (H3K18la) levels (Li et al., 2024). In endothelial cells, HDAC2 acted as a locus-specific eraser of H3K9la, with vascular endothelial growth factor (VEGF)-induced hyperlactylation suppressing HDAC2 transcription to form a positive feedback loop (Fan et al., 2024). Furthermore, HDAC1 itself is subject to regulatory lactylation at K412 in colorectal cancer, which is essential for ferroptosis resistance (Yang Z. et al., 2025). Sirtuin 3 (SIRT3), a well-characterized NAD + -dependent mitochondrial deacetylase, has also been shown to function as a delactylase in several studies. Research indicates that downregulation of SIRT3 leads to the accumulation of H4K12la in renal tubular epithelial cells, which in turn activates runt-related transcription factor 1 (RUNX1) expression and promotes renal fibrosis (Shen et al., 2026). Overexpression of SIRT3 significantly reduces the expression of RUNX1 and α-SMA, further supporting the existence of a SIRT3/H4K12la/RUNX1 regulatory axis in diabetic kidney disease models (Shen et al., 2026).

As a reversible post-translational modification, lactylation is regulated by the “writer-erase” system. Further investigation into this regulatory axis will contribute to a more precise assessment of lactate metabolism and epigenetic regulation in diabetic kidney disease, thereby laying the foundation for the development of biological markers for its diagnosis and prognosis (Li et al., 2024). Beyond these enzymatic regulators, it is important to recognize that lysine lactylation does not function in isolation. It intersects with other post-translational modifications such as ubiquitination, phosphorylation, and small ubiquitin-like modifier (SUMOylation). A recent comprehensive review on SUMOylation has systematically delineated its regulatory effects on protein stability, subcellular localization, transcriptional activity, and signal transduction (Wu et al., 2026). While this framework originates from tumor research, it provides a conceptual springboard for exploring whether lactylation, SUMOylation, and ubiquitination collectively modulate master DKD regulators such as HIF-1α, whose K644 lactylation has been shown to inhibit VHL-mediated ubiquitination (Li et al., 2025). Therefore, investigating the crosstalk among various post-translational modifications (PTMs) in the kidney is critical for achieving a comprehensive understanding of DKD pathogenesis and for designing combination therapies that simultaneously target multiple modification nodes.

As illustrated in the following Figure 3 and Table 1, the hyperglycemic and hypoxic microenvironment in diabetic kidney disease drives metabolic reprogramming and lactate accumulation, which in turn initiates both lactylation-dependent and lactylation-independent pathogenic cascades, ultimately leading to renal injury.

**FIGURE 3:**
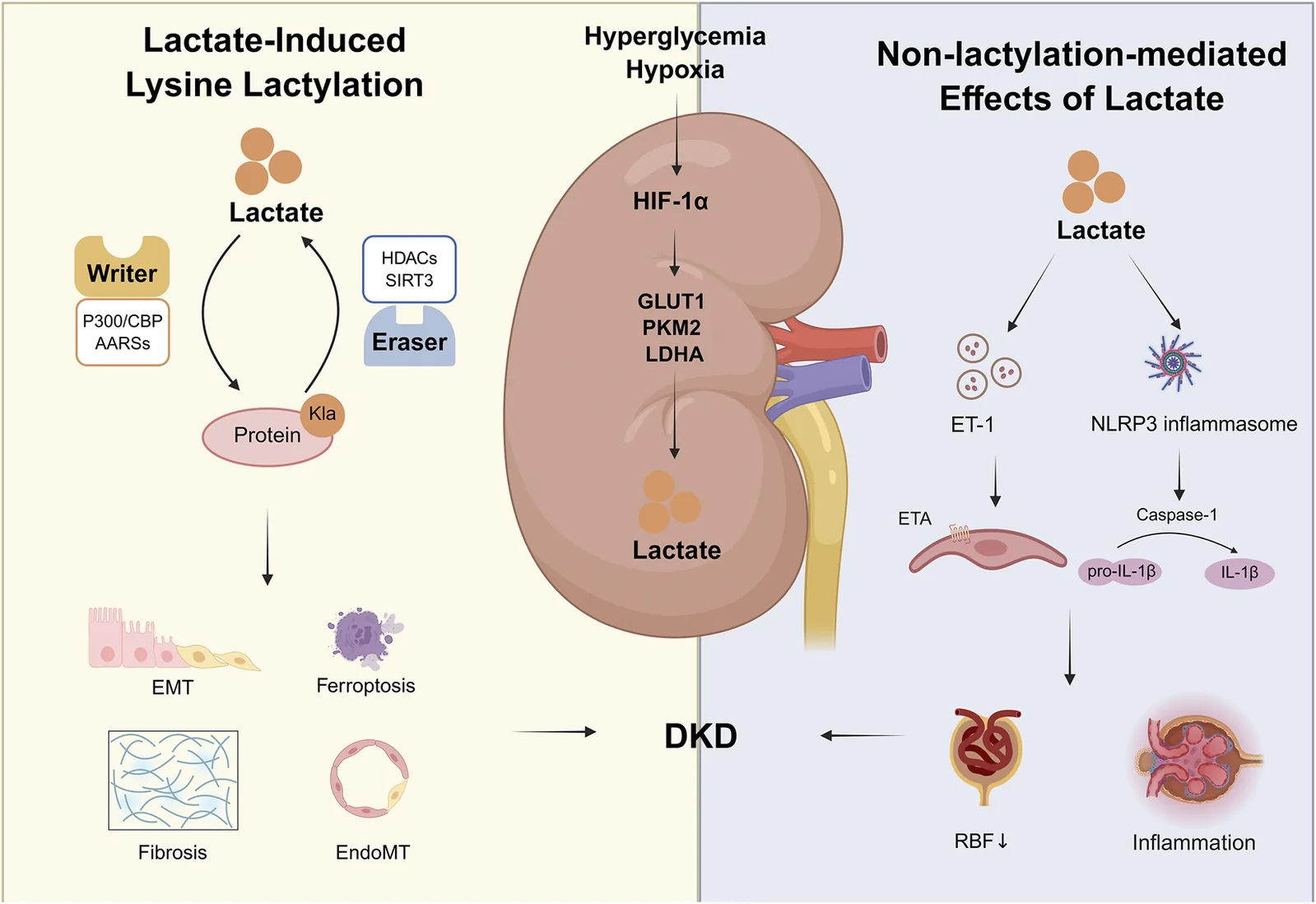
Dual metabolic axes of lactate-mediated injury in DKD. Under chronic hyperglycemia and tissue hypoxia, stabilized HIF-1α orchestrates a metabolic shift toward glycolysis by upregulating glycolytic enzymes (GLUT1, PKM2, LDHA), culminating in massive intracellular and extracellular lactate accumulation. In DKD pathogenesis, lactate exerts its deleterious effects through two interconnected molecular axes: (Left axis: Lactylation-dependent mechanisms.) Elevated lactate serves as a substrate for Kla, a dynamic post-translational modification catalyzed by “writer” complexes (p300/CBP and AARSs) and counteracted by “eraser” enzymes (HDACs and SIRT3). This lactylation-mediated epigenetic remodeling drives pathogenic cellular phenotypes, including EMT, ferroptosis, renal fibrosis, and EndoMT. (Right axis: Non-lactylation-dependent signaling.) Lactate initiates injury through lactate receptor-mediated pathways: extracellularly, lactate activates the GPR81/ET-1 signaling axis, inducing renal vasoconstriction and reducing RBF; intracellularly, lactate triggers NLRP3 inflammasome assembly and caspase-1-dependent (and -independent) pro-IL-1β maturation, amplifying sterile inflammation. These synergistic pathways converge to accelerate DKD progression and renal dysfunction. Abbreviations: AARSs, alanyl-tRNA synthetases; CBP, cAMP response element-binding protein-binding protein; DKD, diabetic kidney disease; EMT, epithelial-mesenchymal transition; EndoMT, endothelial-to-mesenchymal transition; ET-1, endothelin-1; GLUT1, glucose transporter 1; GPR81, G protein-coupled receptor 81; HDACs, histone deacetylases; HIF-1α, hypoxia-inducible factor-1α; IL-1β, interleukin-1β; Kla, lysine lactylation; LDHA, lactate dehydrogenase A; NLRP3, NOD-like receptor family pyrin domain-containing 3; PKM2, pyruvate kinase M2; RBF, renal blood flow; SIRT3, sirtuin 3.

**TABLE 1:** Summary of lactylation writer and eraser enzymes.

Enzyme	Proposed role	Validated substrates	Cell type	Experimental conditions	Evidence
p300/CBP	Writer	H3K9la; H4K12la; HMGB1	Macrophages; microglia; colorectal cancer stem cells	*In vitro* enzymatic assay; pharmacological inhibition	Yang et al. (2022); Deng et al. (2025); Li B. et al. (2026)
AARSs	Writer	H3K18la; STAT1 K193la	DKD tubular cells; endothelial cells	Gene overexpression and knockdown; mutant	Hong et al. (2026)
HDAC1	Eraser	H3K18la; H4K5la; H4K12la	Colorectal cancer stem cells; Hela cells	*In vitro* enzymatic assay; pharmacological inhibition; gene overexpression and knockdown	Moreno-Yruela et al. (2022); Deng et al. (2025)
HDAC2	Eraser	H3K9la; H3K18la; H4K5la	PDAC cells; endothelial cells; Hela cells	*In vitro* enzymatic assay; pharmacological inhibition; gene overexpression and knockdown	Moreno-Yruela et al. (2022); Fan et al. (2024)
HDAC3	Eraser	H3K9la; H3K18la; H4K5la; H4K12la	Hela cells	*In vitro* enzymatic assay; kinetic analysis	Moreno-Yruela et al. (2022)
SIRT3	Eraser	H4K12la	Tubular epithelium cells	Pharmacological inhibition; gene overexpression; NAD^+^ measurement	Shen et al. (2026)

## 5 Lactate-induced lysine lactylation mechanisms in DKD

As previously discussed, in the pathological progression of DKD, lactate functions not only as the end product of aerobic glycolysis but also as a key signaling molecule mediating lactylation (Manoharan et al., 2021). This process links metabolic alterations to epigenetic regulation. lactate-mediated lactylation collectively contributes to the progression of renal injury, ultimately leading to progressive renal function decline, primarily through two core pathways: a feedforward regulatory loop of metabolic reprogramming and immune-inflammatory remodeling.

### 5.1 Feedforward regulatory circuit of lactate-mediated metabolic reprogramming

A feedforward regulatory circuit in metabolic reprogramming promotes the expression of glycolytic genes, leading to progressive lactate accumulation. HIF-1α, a crucial transcription factor regulating glycolytic genes, is stabilized under hypoxic conditions and by ROS. Recent studies indicate that lactate-induced lactylation can further activate HIF-1α via a feedforward mechanism, enhancing the expression of related glycolytic genes (Li et al., 2025). Specifically, lactylation occurs at specific lysine residues on HIF-1α (K644 in mice, and the corresponding K12 in humans and pigs). This modification inhibits the interaction between the VHL protein and hydroxylated HIF-1α, thereby suppressing HIF-1α ubiquitination and subsequent proteasomal degradation (Li et al., 2025). Consequently, the expression of glycolytic genes such as GLUT1, LDHA and Hexokinase 2 (HK2) is upregulated (Li et al., 2025). Existing evidence demonstrates that lactylation of mitochondrial proteins can impair oxidative phosphorylation. Studies show that hypoxia stabilizes the mitochondrial protein lysine transferase alanyl-tRNA synthetase 2 (AARS2), which induces lactylation of pyruvate dehydrogenase E1 subunit alpha 1 (PDHA1) and carnitine palmitoyltransferase 2 (CPT2), limiting the supply of acetyl-CoA and thereby constraining mitochondrial oxidative phosphorylation (Mao et al., 2024). In acute kidney injury (AKI) models, renal lactate accumulation induces lactylation of citrate synthase (CS) at K370. This modification leads to mitochondrial damage, decreased mitochondrial membrane potential (MMP), increased mitochondrial ROS (mtROS) production, inhibition of oxidative phosphorylation, and activation of the NLRP3 inflammasome (Yu et al., 2025). These events collectively contribute to sustained renal inflammation and fibrosis, facilitating the transition from AKI to CKD (Yu et al., 2025).

Research in other diseases further reveals that lactate, as a glycolytic end-product, can act as a metabolic signal to promote the transcriptional activation of stress- and metabolism-related genes via histone lactylation. In pancreatic ductal adenocarcinoma models, lactate-induced H3K18la promotes the transcription of TTK and BUB1B, upregulates p300, and activates LDHA, resulting in progressive lactate accumulation that further enhances histone lactylation (Li et al., 2024). In Alzheimer’s disease (AD) models, metabolic reprogramming has been demonstrated in microglia. Lactate accumulation in microglia elevates H4K12la levels, which enriches at the promoters of glycolytic genes such as PKM2, activates their transcription, and increases glycolytic flux (Pan et al., 2022). Therefore, lactate-induced lactylation may amplify the expression of glycolysis-related genes and reinforce the metabolic feedback loop in DKD, although this hypothesis requires further validation through kidney-specific experiments.

### 5.2 Cell-specific roles of lactate metabolism and lactylation

Beyond exacerbating intracellular metabolic reprogramming through epigenetic modifications-thereby profoundly altering energy metabolism and biosynthetic pathways-lactate-induced lactylation may also induce phenotypic and functional changes in various renal cell types. These changes specifically manifest as podocyte epithelial-mesenchymal transition (EMT) and apoptosis, renal tubular epithelial cell (RTEC) activation and ferroptosis, and glomerular endothelial cell (GEnC) dysfunction and inflammasome activation, as detailed in the following subsections. This complex cascade of cellular events collectively remodels the local renal immune-inflammatory microenvironment, which may lead to sustained and progressive damage to renal structure and function, thereby contributing to the pathological progression of diabetic kidney disease. However, it is important to recognize that renal inflammation in DKD can result from diverse upstream pathways beyond lactate alone.

#### 5.2.1 Podocytes

Podocytes are terminally differentiated cells attached to the outer surface of the glomerular basement membrane and constitute a critical component of the glomerular filtration barrier. Their dysfunction is a major contributor to the progression of diabetic kidney disease. Accumulating evidence indicates that metabolic reprogramming and lactate signaling are key drivers of podocyte injury in diabetes. In mouse podocyte lines exposed to high glucose, prolonged stimulation, upregulated expression of the MCT2, and reduced pyruvate dehydrogenase (PDH) activity led to elevated renal lactate levels. This subsequently reduces the levels of nephrin and Zonula Occludens-1 (ZO-1), increases histone lactylation, and elevates collagen IV and αlpha-smooth muscle actin (α-SMA) expression. These changes may drive the transcriptional reprogramming associated with podocyte EMT, thereby promoting the pathophysiological progression of diabetic kidney disease (Zheng et al., 2025). Furthermore, leucyl-tRNA synthetase 1 (LARS1), an intracellular leucine sensor, regulates cellular autophagy levels via the mechanistic target of rapamycin (mTORC1) signaling pathway. Studies have shown that under high-glucose conditions in patients with diabetic kidney disease, lactate accumulation in podocytes increases lactylation at the K970 site of LARS1. This modification leads to the sustained intracellular accumulation of LARS1 protein, which in turn activates the mTORC1 complex. Persistent mTORC1 activation suppresses autophagic flux, rendering cells unable to clear damaged mitochondria. As mitochondrial function deteriorates, intracellular reactive oxygen species (ROS) levels progressively rise, ultimately triggering podocyte apoptosis and disrupting the glomerular filtration barrier. Consistently, transfection with siRNA to knock down LARS1 significantly improves renal function and alleviates podocyte damage (Fan et al., 2025; Li Z. et al., 2026).

In addition to these lactylation-dependent mechanisms, experiments using podocyte-specific Nlrp3 gain-of-function and loss-of-function mouse models demonstrated that NLRP3 inflammasome activation in podocytes is both sufficient and necessary to drive glomerular sterile inflammation and injury in diabetic kidney disease. Mechanistically, podocyte NLRP3 promotes inflammation through the canonical caspase-1/IL-1β pathway while concurrently suppressing glomerular autophagy via a non-canonical, caspase-1-independent mechanism, thereby aggravating podocyte injury through a non-lactylation mechanism (Shahzad et al., 2022).

In summary, hyperglycemia-driven metabolic reprogramming leads to lactate accumulation in podocytes, which promotes injury through both lactylation-dependent and lactylation-independent mechanisms. On one hand, histone lactylation epigenetically may drive podocyte EMT, downregulating slit diaphragm proteins such as nephrin and ZO-1 while upregulating fibrotic markers including collagen IV and α-SMA. On the other hand, NLRP3 inflammasome activation aggravates podocyte damage via the canonical caspase-1/IL-1β inflammatory pathway and a non-canonical, caspase-1-independent suppression of autophagy, while lactylation of LARS1 at lysine 970 inhibits autophagy and promotes apoptosis through the mTORC1 signaling axis. Collectively, these findings highlight the multifaceted pathogenic roles of lactate in podocyte injury and identify potential therapeutic targets for diabetic kidney disease (Figure 4a).

**FIGURE 4:**
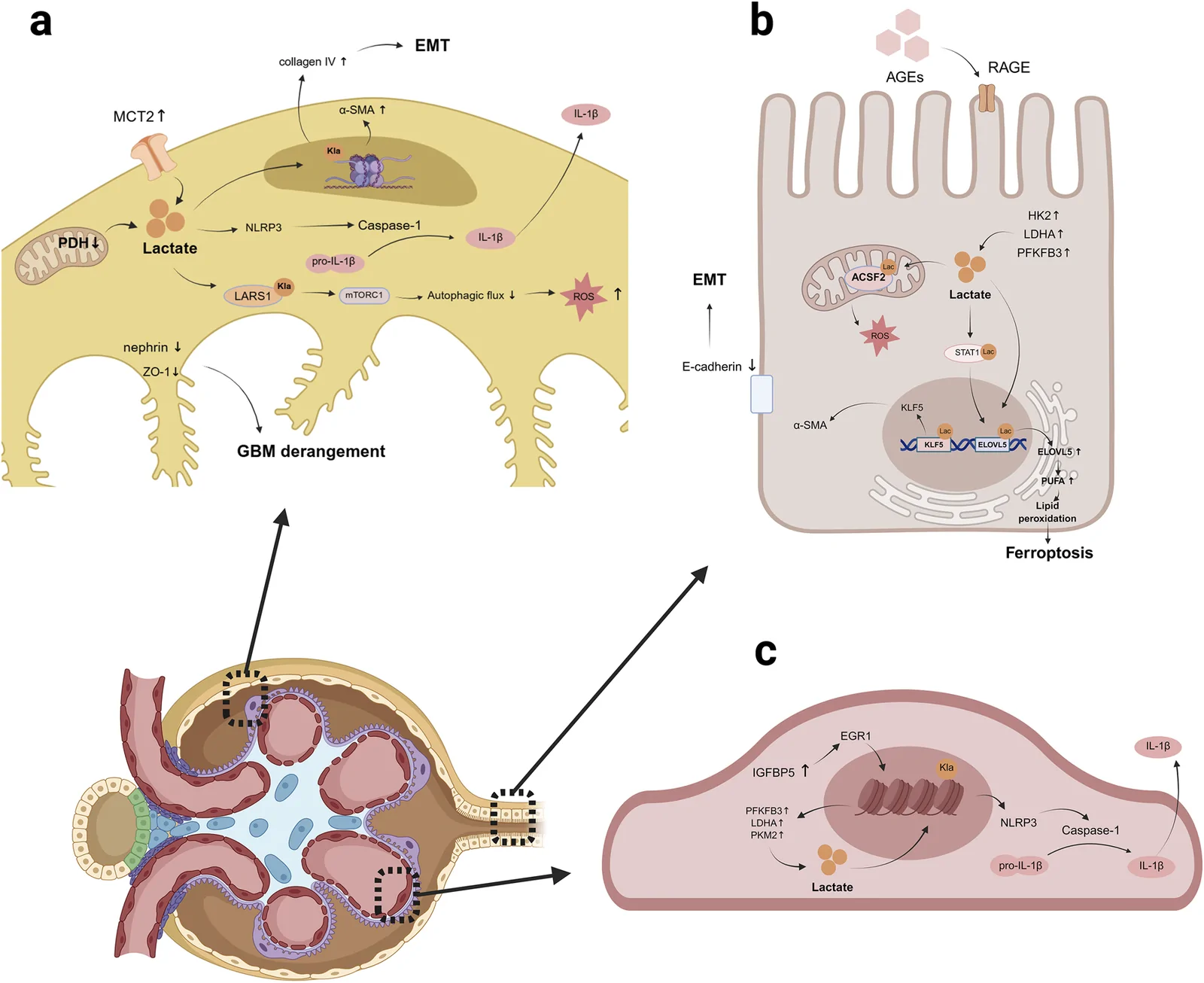
Non-lactylation and lactylation-mediated injury cascades in podocytes, RTECs and GEnCs. **(a)** MCT2-upregulated lactate influx and PDH suppression in podocytes fuel dual pathogenic axes: (1) nuclear histone Kla represses slit diaphragm proteins (nephrin, ZO-1) while inducing α-SMA/collagen IV, promoting podocyte EMT; (2) cytoplasmic LARS1-K970la activates mTORC1, blocks autophagy, and elevates ROS, with ROS further triggering NLRP3/IL-1β inflammation. These converge on foot process effacement and albuminuria. **(b)** AGEs bind RAGE on podocytes, triggering metabolic reprogramming characterized by enhanced MCT2 influx and upregulated glycolysis (HK2, LDHA, PFKFB3), leading to ACSF2-dependent mitochondrial ROS accumulation. This lactate surge drives lysine lactylation modifications on STAT1 and KLF5. Nuclear lactylated STAT1 upregulates ELOVL5, promoting PUFA synthesis and subsequent pro-inflammatory lipid mediator production. Simultaneously, lactylated KLF5 suppresses E-cadherin and induces α-SMA, mediating podocyte EMT and detachment, ultimately accelerating DKD progression. **(c)** IGFBP5-driven EGR1 upregulates glycolytic enzymes (HK2, PFKFB3, LDHA, PKM2), increasing intracellular lactate that serves as the substrate for H3K18la. H3K18la reinforces EGR1 expression, establishing a feed-forward loop, while concurrently promoting NLRP3 inflammasome assembly, caspase-1 activation, and pro-IL-1β maturation, and driving EndoMT. Abbreviations: EMT, epithelial-mesenchymal transition; GBM, glomerular basement membrane; Kla, lysine lactylation; LARS1, leucyl-tRNA synthetase 1; mTORC1, mechanistic target of rapamycin complex 1; MCT2, monocarboxylate transporter 2; PDH, pyruvate dehydrogenase; ROS, reactive oxygen species. ACSF2, acyl-CoA synthetase family member 2; AGEs, advanced glycation end products; ELOVL5, elongase of very long chain fatty acids 5; KLF5, Krüppel-like factor 5; RAGE, receptor for advanced glycation end-products; RTECs, renal tubular epithelial cells; α-SMA, α-smooth muscle actin. EGR1, early growth response protein 1; EndoMT, endothelial-to-mesenchymal transition; GEnCs, glomerular endothelial cells; H3K18la, histone H3 lysine 18 lactylation; IL-1β, interleukin-1β; LDHA, lactate dehydrogenase A; NLRP3, NOD-like receptor family pyrin domain-containing 3; PFKFB3, 6-phosphofructo-2-kinase/fructose-2,6-bisphosphatase 3; PKM2, pyruvate kinase M2.

#### 5.2.2 Renal tubular epithelial cells

In diabetic kidney disease, RTECs undergo significant metabolic and epigenetic reprogramming, accelerating renal fibrosis and functional decline. *In vitro* studies using primary mouse RTECs and *in vivo* diabetic mouse models have shown that advanced glycation end products (AGEs) induce a marked reduction in the levels of Carnitine palmitoyltransferase 1A (CPT1A), CPT2, and Peroxisome proliferator-activated receptor α (PPARα), while upregulating the expression of key glycolytic genes and proteins such as HK2, PFKFB3, and LDHA. This activates aberrant renal glycolysis and leads to renal lactate accumulation. The accumulated lactate is associated with a significant increase in H3K14la levels at the Kruppel-like transcription factor 5 (KLF5) gene promoter, upregulating KLF5 expression and subsequently inhibiting E-cadherin transcription, which may trigger EMT and renal fibrosis. KLF5 knockdown experiments demonstrated that reduced KLF5 expression elevates E-cadherin and ZO-1 levels while decreasing α-SMA and Vimentin expression, further confirming the existence of the AGEs-lactate-H3K14la-KLF5 axis (Zhang et al., 2024).

Lactate-induced lactylation also contributes to mitochondrial damage and ferroptosis in RTECs. Studies indicate that hyperglycemia and lactate accumulation drive extensive lysine lactylation in mouse kidney tissue. Specifically, lactylation of Acyl-CoA synthetase family member 2 (ACSF2) at K182 elevates mitochondrial ROS levels and alters mitochondrial-associated proteins, leading to mitochondrial impairment and dysfunction. Targeted inhibition of ACSF2 restores mitochondrial integrity and alleviates tubular injury, underscoring the critical role of ACSF2 lactylation in linking lactate accumulation to mitochondrial dysfunction (Chen et al., 2024). AARS1 is highly expressed in diabetic kidney disease models and catalyzes lactylation at histone H3K18 and at K193 of STAT1. Both modifications collectively act on the ELOVL fatty acid elongase 5 (ELOVL5) gene promoter to drive its transcription, promoting the synthesis of polyunsaturated fatty acids in the endoplasmic reticulum, which subsequently may induce lipid peroxidation and cellular ferroptosis (Hong et al., 2026).

The lactylation-mediated epigenetic landscape in DKD may be interpreted by master transcription factors, such as SRY-box transcription factor 4 (SOX4) and TEA domain family member 1 (TEAD1), whose roles in DKD remain largely unexplored. Given that lactate accumulation stabilizes HIF-1α and remodels chromatin accessibility via lactylation, it is plausible that the lactate-lactylation axis converges with these transcription factors to orchestrate profibrotic and inflammatory outputs, representing a high-priority mechanistic Frontier (Tran et al., 2025; Du et al., 2026).

Metabolic reprogramming in RTECs may lead to sustained lactate accumulation, which activates lactate-induced lactylation, may drive the activation of fibrotic transcriptional programs, and contributes to persistent mitochondrial dysfunction and cellular ferroptosis. The AGEs-lactate-H3K14la-KLF5 axis, the ACSF2-K182la axis, and the AARS1-H3K18la/STAT1 K193la-ELOVL5 axis are not isolated pathogenic events. Instead, they constitute an interconnected regulatory network through which lactylation may induce RTEC injury, collectively propelling the pathological progression of diabetic kidney disease and providing multiple potential key targets for future therapeutic intervention (Figure 4b).

#### 5.2.3 Glomerular endothelial cells

GEnCs is a central event in the initiation and progression of diabetic kidney disease. Compelling evidence indicates that prolonged hyperglycemia triggers significant metabolic reprogramming in GEnCs. During this process, the expression of insulin-like growth factor-binding protein 5 (IGFBP5) is markedly upregulated, which in turn activates PFKFB3, a key rate-limiting enzyme in the glycolytic pathway and exacerbates the inflammatory response in endothelial cells. These findings initially suggested a potential role for the glycolytic metabolite lactate in mediating GEnC injury (Song et al., 2022). Recent research has further elucidated that IGFBP5 drives substantial lactate production and accumulation within GEnCs by enhancing glycolytic flux via early growth response 1 (EGR1)-mediated PFKFB3 transcription. The accumulated lactate acts as a metabolic signaling molecule that may upregulate the level of H3K18la. This epigenetic modification subsequently may activate the intracellular inflammatory signaling hub, the NLRP3 inflammasome. The sustained activation of the NLRP3 inflammasome, in turn, may drive endothelial-to-mesenchymal transition (EndoMT) in GEnCs, ultimately exacerbating renal fibrotic lesions. Knockdown experiments targeting IGFBP5 have demonstrated that reducing IGFBP5 expression significantly downregulates the activity of key glycolytic enzymes, such as LDHA and PKM2, effectively suppresses aberrant activation of the NLRP3 inflammasome, and thereby markedly attenuates both the EndoMT process and the extent of renal fibrosis (Hu et al., 2025).

It should be noted that, to date, direct evidence linking lactate or lactylation to glomerular endothelial cell (GEnC) injury in diabetic kidney disease remains scarce. Nonetheless, GEnC damage can also arise through alternative, non-lactylation-driven mechanisms, such as lipotoxicity-induced pyroptosis and endothelial dysfunction. For instance, Wu et al. demonstrated that under diabetic conditions, cholesterol overload in GEnCs suppresses ATP-binding cassette transporter A1 (ABCA1) -mediated cholesterol efflux, leading to intracellular cholesterol accumulation, caspase-1-dependent pyroptosis, and subsequent renal injury. Although these findings highlight a parallel route of GEnC injury independent of lactylation, a detailed discussion of lipotoxicity and pyroptosis lies beyond the scope of the present review, which is centered on lactate-driven pathogenic mechanisms (Wu et al., 2024). These pathways are summarized in Figure 4c.

In summary, hyperglycemia-induced metabolic reprogramming serves as a central mechanism contributing to renal cell injury in DKD. Glycolytic activation has been reported in multiple renal cell types, including podocytes, RTECs, and GEnCs under hyperglycemic conditions; however, direct evidence of lactate accumulation and lactylation in GEnCs remains limited and requires further investigation. Functioning as a key metabolic signaling molecule, lactate may drive a series of pathological processes by inducing lactylation modifications on both histones (e.g., H3K14la, H3K18la) and non-histone proteins (e.g., LARS1, ACSF2, STAT1). In podocytes, lactylation is associated with EMT and may suppress autophagy, leading to disruption of the filtration barrier. In RTECs, lactylation is associated with EMT, mitochondrial damage, and ferroptosis via the AGEs-lactate-H3K14la-KLF5 axis, the ACSF2-K182la axis, and the AARS1-H3K18la/STAT1-K193la-ELOVL5 axis. In GEnCs, emerging evidence suggests that lactate modulates H3K18la to activate the NLRP3 inflammasome, potentially exacerbating EndoMT and fibrosis, but the precise role of lactate-induced lactylation in DKD-specific GEnC injury needs to be further validated. These interconnected lactylation-dependent pathways collectively constitute a regulatory network that ultimately results in renal fibrosis and functional decline, and they provide multiple potential therapeutic targets for interventions aimed at lactate metabolism and lactylation modifications (Figure 5).

**FIGURE 5:**
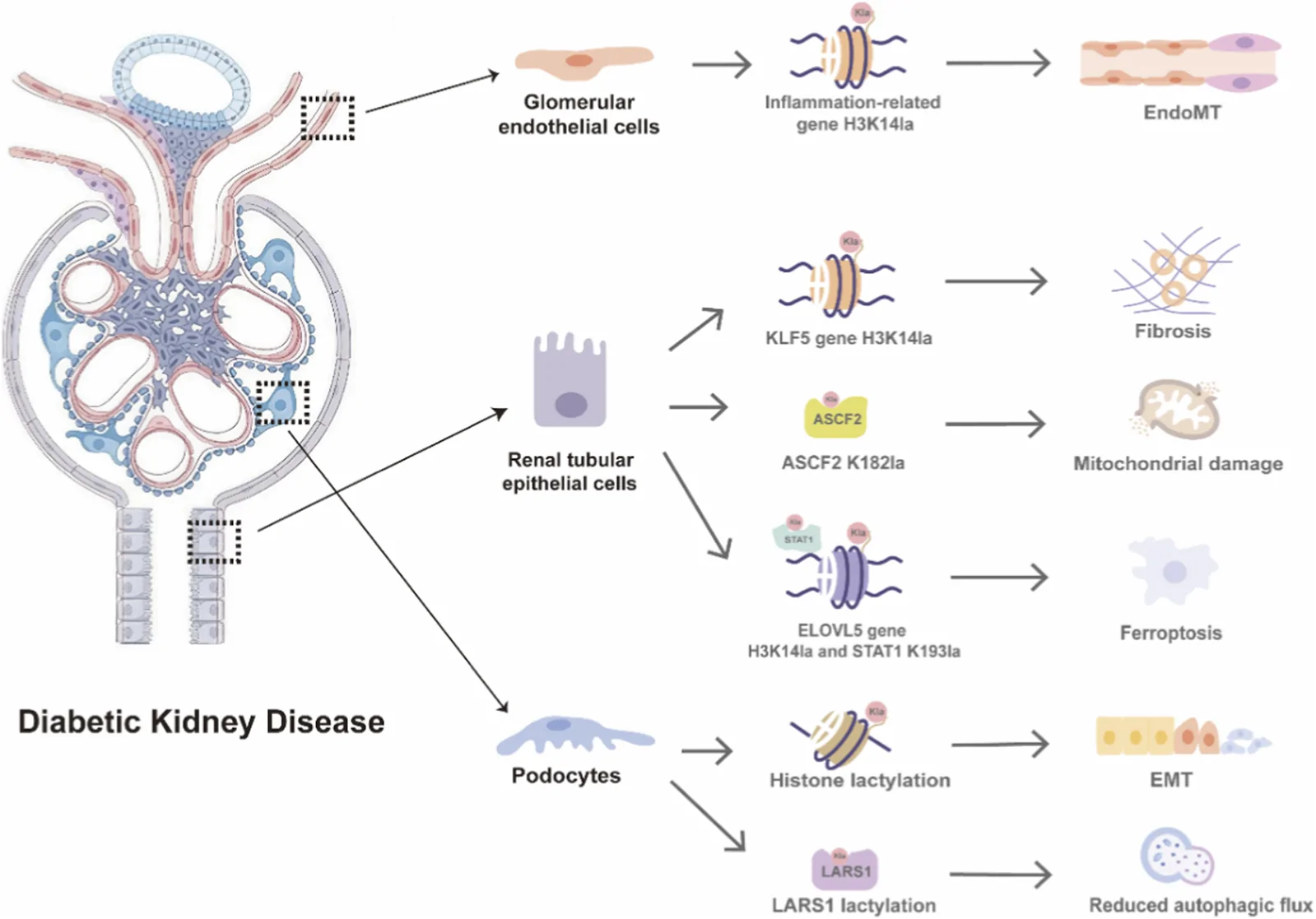
Schematic mechanism of lactate-driven pathogenesis mediated by histone and non-histone lactylation in DKD Lactylation drives pathogenic processes in multiple renal cell types: (i) in podocytes, histone lactylation and LARS1 lactylation inhibit autophagy and induce EMT; (ii) in renal tubular epithelial cells, lactylation of H3K14la at the KLF5 gene fibrosis, K182la at ASCF2 promotes mitochondrial damage, while lactylation at the ELOVL5 gene promoter triggers ferroptosis; (iii) in glomerular endothelial cells, lactylation of H3K14la at inflammation-related genes promotes EndoMT. Abbreviations: EMT, epithelial-mesenchymal transition; EndoMT, endothelial-to-mesenchymal transition; H3K14la, histone H3 lysine 14 lactylation; K193la, lysine 193 lactylation.

## 6 Therapeutic perspectives and challenges

### 6.1 Research progress on targeting lactate-induced lactylation for DKD therapy

Currently, a systematic therapeutic framework for DKD has been progressively established within the global medical community. This framework emphasizes comprehensive lifestyle management as its foundational cornerstone, including strict glycemic and blood pressure control. Built upon this foundation, pharmacotherapies such as renin-angiotensin system (RAS) inhibitors (e.g., ACEI, ARBs) are established as first-line core agents. This integrated therapeutic model aims to effectively decelerate disease progression and achieve comprehensive management through multi-level, multi-target synergistic interventions. While it has succeeded in reducing the risks associated with DKD and its complications to a certain extent, it often fails to halt disease progression entirely (Elendu et al., 2023). Therefore, exploring novel molecular pathways associated with diabetic kidney disease and identifying potential therapeutic targets to prevent or mitigate renal dysfunction is of paramount importance.

Research on the emerging mechanism of lactate-induced lactylation has infused new vitality into targeted therapy for DKD. Theoretically, therapeutic agents could be designed to target glucose uptake, glycolytic pathways, and the lactate-induced lactylation signaling axis. However, current research on such targeted drugs is extremely limited, with most interventions aimed at lactate-induced lactylation remaining confined to *in vitro* cellular studies and animal models.

SGLT2i are a new generation of glucose-lowering agents that reduce glucose reabsorption in the renal proximal tubule, playing a significant role in glycemic control and cardiovascular disease prevention (Tentolouris et al., 2019). As research on SGLT2i deepens, their benefits have extended beyond traditional glucose-lowering effects. Accumulating evidence indicates that SGLT2 inhibitors can reduce albuminuria, significantly mitigate complications associated with declining glomerular filtration rate (GFR), and slow the pathological progression of renal dysfunction (Nobayashi et al., 2026). Current international guidelines have now positioned SGLT2i as first-line therapy for diabetic kidney disease (Rossing et al., 2022). Studies suggest that SGLT2i may confer renoprotection, in part, by rectifying the dysregulated HIF signaling network in diabetic kidney disease (Nair et al., 2026). As previously discussed, HIF-1α is a key transcription factor linking the diabetic renal milieu to metabolic reprogramming. This suggests that SGLT2i might exert their protective effects by inhibiting glucose uptake, lactate production, and subsequent lactylation, though this hypothesis requires further validation in dedicated diabetic kidney disease models.

Traditional Chinese Medicine has attracted research attention for its potential to modulate disease progression. Preclinical evidence from animal models indicates that several herbal extracts can alleviate pathological processes by targeting lactate-mediated lactylation. For instance, in xenograft models of hepatocellular carcinoma, herbal compounds including demethylzeylasteral (DML) and royal jelly acid (RJA) disrupt glycolytic networks through inhibition of key enzymes such as LDHA and PKM2. This subsequently suppresses lactate production and reduces lactylation at histone sites including H3K9 and H3K18 (Qiu and Shao, 2025). In prostate cancer animal models, evodiamine has been shown to inhibit HIF-1α activation and H3K18la expression, increase Semaphorin 3A (Sema3A) levels, and consequently impede vascular endothelial growth factor A (VEGFA)-induced cell proliferation and PD-L1 expression (Yu et al., 2023). Furthermore, Gegen Qinlian Decoction can suppress lactylation at specific histone sites such as H3K18la, H3K23la, H4K8la, and H4K12la, exerting inhibitory effects on inflammation and oxidative stress in murine models of ulcerative colitis (Xu et al., 2024). Notably, current evidence supporting the regulatory effects of Traditional Chinese Medicine on the lactate-lactylation axis remains largely at the preclinical stage, and relevant studies conducted specifically in the context of DKD are extremely limited. Future investigations should be performed in well-established DKD animal models, and in-depth studies are warranted to characterize bioactive constituents, pharmacokinetic profiles, and potential drug interactions of these herbal agents (Figure 6).

**FIGURE 6:**
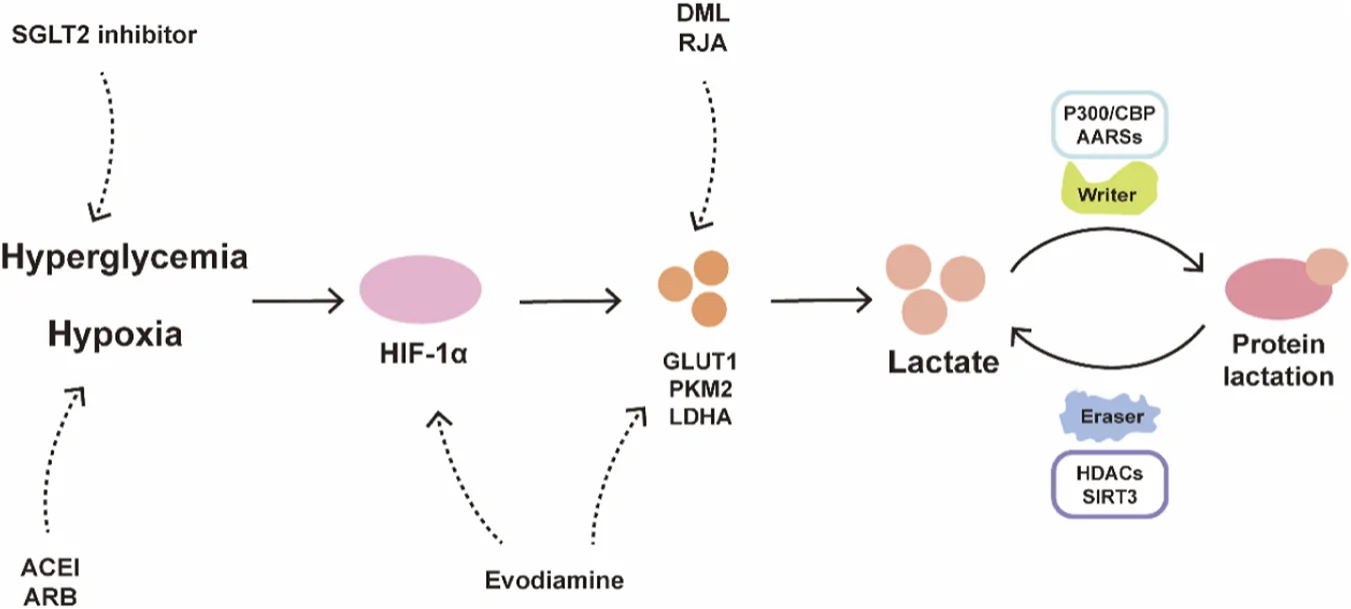
Therapeutic strategies targeting the lactate-lactylation axis in diabetic kidney disease. The schematic depicts the pathological cascade from hyperglycemia/hypoxia to lactate accumulation and lactylation, with corresponding therapeutic interventions indicated at each actionable node. ACEI/ARBs serve as first-line foundational therapy. SGLT2 inhibitors attenuate glucose reabsorption. The traditional Chinese medicine components DML and RJA inhibit key glycolytic enzymes LDHA and PKM2, thereby reducing lactate production. Evodiamine suppresses HIF-1α activation and inhibits key glycolytic enzymes. Abbreviations: ACEI, angiotensin-converting enzyme inhibitor; ARB, angiotensin II receptor blocker; DML, demethylzeylasteral; HIF-1α, hypoxia-inducible factor-1α; GLUT1, glucose transporter 1; LDHA, lactate dehydrogenase A; PKM2, pyruvate kinase M2; RJA, royal jelly acid; SGLT2, sodium-glucose cotransporter 2.

### 6.2 From bench to bedside: clinical translation of lactate-lactylation biomarkers

The ultimate goal of lactate-induced lysine lactylation research is to serve clinical needs. While the translation of lactate and lactylation-related molecules into clinically applicable biomarkers for DKD holds substantial diagnostic value, achieving this goal necessitates a systematic framework. Recent efforts have established a comprehensive “Human Biomarker Navigator” that categorizes biomarkers across nine physiological systems into hierarchical levels - core hubs, regional hubs, and effector nodes - based on their biological scope and signal propagation radius (Li M-Y. et al., 2026). Within this framework, lactate itself may function as a core hub biomarker reflecting systemic metabolic-inflammatory burden, while writers (p300/CBP, AARSs) and erasers (HDACs, SIRT3) could serve as regional hub biomarkers that link epigenetic remodeling to tissue-specific injury; specific lactylation sites such as LARS1-K970la, ACSF2-K182la, and STAT1-K193la may represent effector-node biomarkers that directly quantify organ-level pathology in distinct renal cell types.

To advance these candidate biomarkers toward clinical application, several critical issues must be addressed. First, independent cohort validation is essential to confirm the reproducibility and generalizability of findings beyond the initial discovery populations. Second, rigorous adjustment for confounding factors, including age, sex, body mass index, renal function, and comorbid conditions is necessary to establish true disease-specific associations. Third, standardization of detection methods is required, encompassing pre-analytical sample handling, assay protocols, and inter-laboratory calibration, to ensure measurement reliability and cross-study comparability. Fourth, the diagnostic and prognostic performance of lactate and lactylation-related markers must be systematically evaluated against established clinical benchmarks, particularly urinary albumin-to-creatinine ratio (UACR) and estimated glomerular filtration rate (eGFR), to determine their incremental value in risk stratification and disease monitoring. Importantly, evidence from chronic kidney injury models has demonstrated that urinary kidney injury molecule-1 (KIM-1) and neutrophil gelatinase-associated lipocalin (NGAL) can detect tubular injury 2–4 weeks earlier than serum creatinine elevation (Li M-Y. et al., 2026), providing a reference benchmark for assessing the temporal sensitivity of lactylation-based markers. Through such approaches, the transition from single-marker testing to dynamic, system-level health monitoring for DKD patients may ultimately be achieved.

## 7 Summary

DKD remains a formidable clinical challenge, characterized by persistent microvascular injury that current therapies fail to fully arrest. This review highlights the lactate-lactylation axis as a potential nexus where metabolic dysregulation, epigenetic remodeling, and immune-inflammatory signaling converge to contribute to DKD pathogenesis. It is important to emphasize that DKD is a multifactorial disease, and renal inflammation can result from diverse upstream pathways.

We propose that lactate functions not merely as a metabolic byproduct, but as a potent signaling molecule. Through lysine lactylation, it establishes a self-amplifying feedforward loop—most notably via HIF-1α stabilization—that perpetuates renal injury across podocytes, tubular epithelial cells, and glomerular endothelial cells. This metabolic-epigenetic crosstalk, governed by a dynamic interplay of “writers” and “erasers,” serves as a key contributor to progressive fibrosis and renal failure, although other drivers independent of lactate also exist.

Therapeutically, targeting the lactate-lactylation axis offers a promising therapeutic avenue. While emerging evidence suggests that SGLT2i and certain traditional medicinal compounds may exert renoprotective effects by modulating this pathway, significant work remains. Future efforts should focus on validating key lactylation nodes in DKD-specific models, developing targeted interventions, and establishing lactate/lactylation-based biomarkers for diagnosis and treatment.

## References

[B1] Al-Masri S. Coelho J. N. Thomas L. (2026). Molecular pathways and emerging therapeutic targets in the pathogenesis of diabetic kidney disease. Front. Physiol. 17, 1747053. 10.3389/fphys.2026.1747053 41797844 PMC12960186

[B2] Alicic R. Z. Rooney M. T. Tuttle K. R. (2017). Diabetic kidney disease: challenges, progress, and possibilities. Clin. J. Am. Soc. Nephrol. 12 (12), 2032–2045. 10.2215/CJN.11491116 28522654 PMC5718284

[B3] An C. Jiao B. Du H. Tran M. Zhou D. Wang Y. (2022). Myeloid PTEN deficiency aggravates renal inflammation and fibrosis in angiotensin II-induced hypertension. J. Cell. Physiol. 237 (1), 983–991. 10.1002/jcp.30574 34515350 PMC8810675

[B4] An C. Jiao B. Du H. Tran M. Song B. Wang P. (2023). Jumonji domain-containing protein-3 (JMJD3) promotes myeloid fibroblast activation and macrophage polarization in kidney fibrosis. Br. J. Pharmacol. 180 (17), 2250–2265. 10.1111/bph.16096 37076137 PMC12641593

[B5] Azushima K. Kovalik J. Yamaji T. Ching J. Chng T. W. Guo J. (2023). Abnormal lactate metabolism is linked to albuminuria and kidney injury in diabetic nephropathy. Kidney Int. 104 (6), 1135–1149. 10.1016/j.kint.2023.08.006 37843477

[B6] Bai Y. Chi K. Zhao D. Shen W. Liu R. Hao J. (2024). Identification of functional heterogeneity of immune cells and tubular-immune cellular interplay action in diabetic kidney disease. J. Transl. Int. Med. 12 (4), 395–405. 10.2478/jtim-2023-0130 39360161 PMC11444470

[B7] Becker H. M. Mohebbi N. Perna A. Ganapathy V. Capasso G. Wagner C. A. (2010). Localization of members of MCT monocarboxylate transporter family Slc16 in the kidney and regulation during metabolic acidosis. Am. J. Physiol-Ren. Physiol. 299 (1), F141–F154. 10.1152/ajprenal.00488.2009 20392797

[B8] Benz P. S. Fan X. Wüthrich R. P. (1996). Enhanced tubular epithelial CD44 expression in MRL-lpr lupus nephritis. Kidney Int. 50 (1), 156–163. 10.1038/ki.1996.298 8807584

[B9] Bloomgarden Z. T. (2005). Diabetic nephropathy. Diabetes Care 28 (3), 745–751. 10.2337/diacare.28.3.745 15735220

[B10] Chen J. Feng Q. Qiao Y. Pan S. Liang L. Liu Y. (2024). ACSF2 and lysine lactylation contribute to renal tubule injury in diabetes. Diabetologia 67 (7), 1429–1443. 10.1007/s00125-024-06156-x 38676722

[B11] Cui C. Fu N. Tao S. Chen J. Shang Y. Peng X. (2026). Dapagliflozin may attenuate podocyte injury in diabetic kidney disease through inhibition of lactate-mediated H4K12 lactylation. Kidney Blood Press Res. 51 (1), 394–408. 10.1159/000552052 42030222 PMC13299129

[B12] Darshi M. Kugathasan L. Maity S. Sridhar V. S. Fernandez R. Limonte C. P. (2024). Glycolytic lactate in diabetic kidney disease. JCI Insight 9 (11), e168825. 10.1172/jci.insight.168825 38855868 PMC11382878

[B13] Deng J. Li Y. Yin L. Liu S. Li Y. Liao W. (2025). Histone lactylation enhances GCLC expression and thus promotes chemoresistance of colorectal cancer stem cells through inhibiting ferroptosis. Cell. Death Dis. 16 (1), 193. 10.1038/s41419-025-07498-z 40113760 PMC11926133

[B14] Dong L. Xie J. Tao M. Liu S. Lu Y. Wu T. (2026). Lactylation-dependent DDX18 nucleolar escape regulates CD44 mRNA stability to drive fibrotic progression in kidney injury. J. Clin. Invest. 136, e201848. 10.1172/JCI201848 42593860 PMC13626828

[B15] Du H. Jiao B. Xing J. Yang D. Tran M. Wang P. (2026). Critical role of transcription factor SOX4 in tubular epithelial cell dedifferentiation and fibroblast activation during kidney fibrosis. Kidney Int. 109 (1), 101–118. 10.1016/j.kint.2025.08.030 41005570 PMC12548802

[B16] Elendu C. John Okah M. Fiemotongha K. D. J. Adeyemo B. I. Bassey B. N. Omeludike E. K. (2023). Comprehensive advancements in the prevention and treatment of diabetic nephropathy: a narrative review. Med. Baltim. 102 (40), e35397. 10.1097/MD.0000000000035397 PMC10553077 37800812

[B17] Fan W. Zeng S. Wang X. Wang G. Liao D. Li R. (2024). A feedback loop driven by H3K9 lactylation and HDAC2 in endothelial cells regulates VEGF-induced angiogenesis. Genome Biol. 25 (1), 165. 10.1186/s13059-024-03308-5 38918851 PMC11197246

[B18] Fan Z. Zhang Y. Yuan L. Gao Y. Tian X. Tian J. (2025). LARS1 lactylation inhibits autophagy by activating mTORC1 to promote podocytes injury in diabetic kidney disease. Cell. Signal 134, 111955. 10.1016/j.cellsig.2025.111955 40545110

[B19] Gong N. Wang W. Fu Y. Zheng X. Guo X. Chen Y. (2025). The crucial role of metabolic reprogramming in driving macrophage conversion in kidney disease. Cell. Mol. Biol. Lett. 30 (1), 72. 10.1186/s11658-025-00746-2 40524149 PMC12172235

[B20] Gouchoe D. A. Zhang Z. Kim J. Lee Y. G. Whitson B. A. Zhu H. (2024). Improving lung allograft function in the early post-operative period through the inhibition of pyroptosis. Med. Rev. (2021) 4 (5), 384–394. 10.1515/mr-2023-0066 39444796 PMC11495470

[B21] Halestrap A. P. (2013). Monocarboxylic acid transport. Compr. Physiol. 3 (4), 1611–1643. 10.1002/cphy.c130008 24265240

[B22] Hesp A. C. Schaub J. A. Prasad P. V. Vallon V. Laverman G. D. Bjornstad P. (2020). The role of renal hypoxia in the pathogenesis of diabetic kidney disease: a promising target for newer renoprotective agents including SGLT2 inhibitors? Kidney Int. 98 (3), 579–589. 10.1016/j.kint.2020.02.041 32739206 PMC8397597

[B23] Hong J. Xu H. Yu L. Yu Z. Chen X. Meng Z. (2026). AARS1-mediated lactylation of H3K18 and STAT1 promotes ferroptosis in diabetic nephropathy. Cell. Death Differ. 33 (3), 589–604. 10.1038/s41418-025-01587-4 40987895 PMC13036035

[B24] Hu X. Chen W. Yang M. Li M. Li X. Ouyang S. (2025). IGFBP5 promotes EndoMT and renal fibrosis through H3K18 lactylation in diabetic nephropathy. Cell. Mol. Life Sci. 82 (1), 215. 10.1007/s00018-025-05718-5 40423799 PMC12116956

[B25] Iyer N. V. Leung S. W. Semenza G. L. (1998). The human hypoxia-inducible factor 1alpha gene: HIF1A structure and evolutionary conservation. Genomics 52 (2), 159–165. 10.1006/geno.1998.5416 9782081

[B26] Jiang N. Zhao H. Han Y. Li L. Xiong S. Zeng L. (2020). HIF-1α ameliorates tubular injury in diabetic nephropathy via HO-1-mediated control of mitochondrial dynamics. Cell. Prolif. 53 (11), e12909. 10.1111/cpr.12909 32975326 PMC7653251

[B27] Jiao B. An C. Du H. Tran M. Wang P. Zhou D. (2021). STAT6 deficiency attenuates myeloid fibroblast activation and macrophage polarization in experimental folic acid nephropathy. Cells 10 (11), 3057. 10.3390/cells10113057 34831280 PMC8623460

[B28] Jiao B. An C. Tran M. Du H. Wang P. Zhou D. (2021). Pharmacological inhibition of STAT6 ameliorates myeloid fibroblast activation and alternative macrophage polarization in renal fibrosis. Front. Immunol. 12, 735014. 10.3389/fimmu.2021.735014 34512669 PMC8426438

[B29] Jones N. K. Stewart K. Czopek A. Menzies R. I. Thomson A. Moran C. M. (2020). Endothelin-1 mediates the systemic and renal hemodynamic effects of GPR81 activation. Hypertension 75 (5), 1213–1222. 10.1161/HYPERTENSIONAHA.119.14308 32200679 PMC7176350

[B30] Jung C. Yoo T. (2022). Pathophysiologic mechanisms and potential biomarkers in diabetic kidney disease. Diabetes Metab. J. 46 (2), 181–197. 10.4093/dmj.2021.0329 35385633 PMC8987689

[B31] Li F. Si W. Xia L. Yin D. Wei T. Tao M. (2024). Positive feedback regulation between glycolysis and histone lactylation drives oncogenesis in pancreatic ductal adenocarcinoma. Mol. Cancer 23 (1), 90. 10.1186/s12943-024-02008-9 38711083 PMC11071201

[B32] Li C. Fu C. Zhou W. Li H. Liu Z. Wu G. (2025). Lactylation modification of HIF-1α enhances its stability by blocking VHL recognition. Cell. Commun. Signal 23 (1), 364. 10.1186/s12964-025-02366-x 40760493 PMC12323271

[B33] Li B. Xu K. Yu J. (2026). Lactate modulates microglial M2 polarization via H3K9 lactylation in ischemic stroke. J. Transl. Int. Med. 14 (2), 276–293. 10.1515/jtim-2026-0010 42046804 PMC13110458

[B34] Li M.-Y. Zhang Q. Wang Z. Gui Z. Liu J. Li J. (2026). Human biomarker navigator. iMeta n/a, e70157. 10.1002/imt2.70157 42630486 PMC13494139

[B35] Li Z. Wang Y. Fang D. Ye W. Zhang X. Chen Z. (2026). Role of metabolic reprogramming and lactylation in diabetic nephropathy: molecular mechanisms and therapeutic prospects - a narrative review. Front. Endocrinol. (Lausanne) 17, 1770531. 10.3389/fendo.2026.1770531 41767381 PMC12945752

[B36] Lin H. Lin H. Tsai M. Chen Y. Bao B. Tsai K. (2026). Lactic acid drives NLRP3 inflammasome activation and caspase-1-like cytokine cleavage via intracellular acidification. Cell. Death Dis. 17 (1), 450. 10.1038/s41419-026-08708-y 41932879 PMC13172327

[B37] Liu C. Wu J. Zhu J. Kuei C. Yu J. Shelton J. (2009). Lactate inhibits lipolysis in fat cells through activation of an orphan G-protein-coupled receptor, GPR81. J. Biol. Chem. 284 (5), 2811–2822. 10.1074/jbc.M806409200 19047060

[B38] Liu E. Weng C. Yan C. Zhu X. Li X. Liu M. (2025). Lactate programs PBX1 lactylation and mesangial proliferation in lupus nephritis. JCI Insight 10 (11), e190838. 10.1172/jci.insight.190838 40299657 PMC12220973

[B39] Manoharan I. Prasad P. D. Thangaraju M. Manicassamy S. (2021). Lactate-dependent regulation of immune responses by dendritic cells and macrophages. Front. Immunol. 12, 691134. 10.3389/fimmu.2021.691134 34394085 PMC8358770

[B40] Mao Y. Zhang J. Zhou Q. He X. Zheng Z. Wei Y. (2024). Hypoxia induces mitochondrial protein lactylation to limit oxidative phosphorylation. Cell. Res. 34 (1), 13–30. 10.1038/s41422-023-00864-6 38163844 PMC10770133

[B41] Moreno-Yruela C. Zhang D. Wei W. Bæk M. Liu W. Gao J. (2022). Class I histone deacetylases (HDAC1-3) are histone lysine delactylases. Sci. Adv. 8 (3), eabi6696. 10.1126/sciadv.abi6696 35044827 PMC8769552

[B42] Nair V. Minakawa A. Smith C. Berthier C. C. Hartman J. Menon R. (2026). Hypoxia inducible factor network reflects kidney disease progression in diabetes and sodium-glucose co-transporters inhibition. Signal Transduct. Target Ther. 11 (1), 144. 10.1038/s41392-026-02653-0 42009641 PMC13096240

[B43] Nobayashi H. Satoh M. Hirose T. Nakayama S. Iwabe Y. Hashimoto H. (2026). Comparison of SGLT2 inhibitors for new-onset proteinuria risk in patients with type 2 diabetes and preserved kidney function. Diabetes Obes. Metab. 28 (5), 4314–4325. 10.1111/dom.70625 41804189 PMC13071193

[B44] Pan R. He L. Zhang J. Liu X. Liao Y. Gao J. (2022). Positive feedback regulation of microglial glucose metabolism by histone H4 lysine 12 lactylation in Alzheimer's disease. Cell. Metab. 34 (4), 634–648. 10.1016/j.cmet.2022.02.013 35303422

[B45] Qiu Y. Shao X. (2025). Histone lactylation in diseases: regulation by traditional Chinese medicine and therapeutic implications. Drug Des. Devel Ther. 19, 6435–6459. 10.2147/DDDT.S524008 40756263 PMC12317698

[B46] Rodríguez-Prados J. Través P. G. Cuenca J. Rico D. Aragonés J. Martín-Sanz P. (2010). Substrate fate in activated macrophages: a comparison between innate, classic, and alternative activation. J. Immunol. 185 (1), 605–614. 10.4049/jimmunol.0901698 20498354

[B47] Rossing P. Caramori M. L. Chan J. C. N. Heerspink H. J. L. Hurst C. Khunti K. (2022). Executive summary of the KDIGO 2022 Clinical Practice Guideline for Diabetes Management in Chronic Kidney Disease: an update based on rapidly emerging new evidence. Kidney Int. 102 (5), 990–999. 10.1016/j.kint.2022.06.013 36272755

[B48] Ruiz-García A. Monsalve E. Novellasdemunt L. Navarro-Sabaté A. Manzano A. Rivero S. (2011). Cooperation of adenosine with macrophage Toll-4 receptor agonists leads to increased glycolytic flux through the enhanced expression of PFKFB3 gene. J. Biol. Chem. 286 (22), 19247–19258. 10.1074/jbc.M110.190298 21464136 PMC3103303

[B49] Shahzad K. Fatima S. Khawaja H. Elwakiel A. Gadi I. Ambreen S. (2022). Podocyte-specific Nlrp3 inflammasome activation promotes diabetic kidney disease. Kidney Int. 102 (4), 766–779. 10.1016/j.kint.2022.06.010 35779608

[B50] Shen S. Ying C. Fu X. Zeng X. Guo X. Wu H. (2026). Delactylase effects of SIRT3 on a positive feedback loop involving the RUNX1-glycolysis-histone lactylation in diabetic kidney disease. Int. J. Biol. Sci. 22 (4), 1775–1792. 10.7150/ijbs.126011 41694585 PMC12905586

[B51] Song C. Wang S. Fu Z. Chi K. Geng X. Liu C. (2022). IGFBP5 promotes diabetic kidney disease progression by enhancing PFKFB3-mediated endothelial glycolysis. Cell. Death Dis. 13 (4), 340. 10.1038/s41419-022-04803-y 35418167 PMC9007962

[B52] Stanigut A. M. Pana C. Enciu M. Deacu M. Cimpineanu B. Tuta L. A. (2022). Hypoxia-Inducible factors and diabetic kidney disease-how deep can we go? Int. J. Mol. Sci. 23 (18), 10413. 10.3390/ijms231810413 36142323 PMC9499602

[B53] Tentolouris A. Vlachakis P. Tzeravini E. Eleftheriadou I. Tentolouris N. (2019). SGLT2 inhibitors: a review of their antidiabetic and cardioprotective effects. Int. J. Environ. Res. Public Health 16 (16). 10.3390/ijerph16162965 31426529 PMC6720282

[B54] Tiwari R. Sharma R. Rajendran G. Borkowski G. S. An S. Y. Schonfeld M. (2024). Postischemic inactivation of HIF prolyl hydroxylases in endothelium promotes maladaptive kidney repair by inducing glycolysis. J. Clin. Invest. 135 (3), e176207. 10.1172/JCI176207 39621585 PMC11785929

[B55] Tran M. Jiao B. Du H. Zhou D. Yechoor V. Wang Y. (2025). TEAD1 prevents necroptosis and inflammation in cisplatin-induced acute kidney injury through maintaining mitochondrial function. Int. J. Biol. Sci. 21 (2), 565–578. 10.7150/ijbs.104335 39781453 PMC11705647

[B56] Wang M. Pang Y. Guo Y. Tian L. Liu Y. Shen C. (2022). Metabolic reprogramming: a novel therapeutic target in diabetic kidney disease. Front. Pharmacol. 13, 970601. 10.3389/fphar.2022.970601 36120335 PMC9479190

[B57] Wang Y. Huang B. Wei X. Guan Y. Li L. Zheng Y. (2026). Gut microbiota metabolic reprogramming drives the development of metabolic diseases in the host. Gut Microbes 18 (1), 2644681. 10.1080/19490976.2026.2644681 PMC12990950 41830551

[B58] Warburg O. Wind F. Negelein E. (1927). The metabolism of tumors in the body. J. Gen. Physiol. 8 (6), 519–530. 10.1085/jgp.8.6.519 19872213 PMC2140820

[B59] Wei X. Long M. Yu J. Du Y. (2025). The lactate-lactylation axis in renal fibrosis: potential mechanisms in diabetic kidney disease. Ann. Med. 57 (1), 2587326. 10.1080/07853890.2025.2587326 PMC12621351 41236504

[B60] Wu T. Ding L. Andoh V. Zhang J. Chen L. (2023). The mechanism of hyperglycemia-induced renal cell injury in diabetic nephropathy disease: an update. Life (Basel) 13 (2), 539. 10.3390/life13020539 36836895 PMC9967500

[B61] Wu W. Wang Y. Shao X. Huang S. Wang J. Zhou S. (2024). GLP-1RA improves diabetic renal injury by alleviating glomerular endothelial cells pyrotosis via RXRα/circ8411/miR-23a-5p/ABCA1 pathway. PLoS One 19 (12), e314628. 10.1371/journal.pone.0314628 39621727 PMC11611192

[B62] Wu Y. Chen Z. Hu Y. Zengin G. Zhang Q. Li M. (2026). SUMOylation regulates tumorigenesis and progression: molecular mechanisms and therapeutic applications. Interdiscip. Med. 4 (3), e70120. 10.1002/inmd.70120

[B63] Xu Z. Shan S. Cai E. Wu Y. (2024). Gegen Qinlian decoction inhibited M1 macrophage polarization and ulcerative colitis progression through regulating histone lactylation. Tissue Cell. 89, 102468. 10.1016/j.tice.2024.102468 39003913

[B64] Yang K. Xu J. Fan M. Tu F. Wang X. Ha T. (2020). Lactate suppresses macrophage pro-inflammatory response to LPS stimulation by inhibition of YAP and NF-κB activation via GPR81-Mediated signaling. Front. Immunol. 11, 587913. 10.3389/fimmu.2020.587913 33123172 PMC7573489

[B65] Yang K. Fan M. Wang X. Xu J. Wang Y. Tu F. (2022). Lactate promotes macrophage HMGB1 lactylation, acetylation, and exosomal release in polymicrobial sepsis. Cell. Death Differ. 29 (1), 133–146. 10.1038/s41418-021-00841-9 34363018 PMC8738735

[B66] Yang Q. Huo E. Cai Y. Zhang Z. Dong C. Asara J. M. (2023). Myeloid PFKFB3-mediated glycolysis promotes kidney fibrosis. Front. Immunol. 14, 1259434. 10.3389/fimmu.2023.1259434 38035106 PMC10687406

[B67] Yang G. Liu X. Li Y. Li L. Xiang J. Liang Z. (2025a). TRIM65 as a key regulator of ferroptosis and glycolysis in lactate-driven renal tubular injury and diabetic kidney disease. Cell. Rep. 44 (8), 116091. 10.1016/j.celrep.2025.116091 40748757

[B68] Yang Z. Su W. Zhang Q. Niu L. Feng B. Zhang Y. (2025b). Lactylation of HDAC1 confers resistance to ferroptosis in colorectal cancer. Adv. Sci. (Weinh) 12 (12), e2408845. 10.1002/advs.202408845 PMC11947995 39888307

[B69] Yao L. Liang X. Qiao Y. Chen B. Wang P. Liu Z. (2022). Mitochondrial dysfunction in diabetic tubulopathy. Metabolism 131, 155195. 10.1016/j.metabol.2022.155195 35358497

[B70] Yu Y. Huang X. Liang C. Zhang P. (2023). Evodiamine impairs HIF1A histone lactylation to inhibit Sema3A-mediated angiogenesis and PD-L1 by inducing ferroptosis in prostate cancer. Eur. J. Pharmacol. 957, 176007. 10.1016/j.ejphar.2023.176007 37611839

[B71] Yu F. Meng Y. Wang X. Chen J. He J. Yi X. (2025). Protein lactylation of citrate synthase promotes the AKI-CKD transition by activating the NLRP3 inflammasome. Cell. Rep. 44 (8), 116084. 10.1016/j.celrep.2025.116084 40748753

[B72] Zhang X. Chen J. Lin R. Huang Y. Wang Z. Xu S. (2024). Lactate drives epithelial-mesenchymal transition in diabetic kidney disease via the H3K14la/KLF5 pathway. Redox Biol. 75, 103246. 10.1016/j.redox.2024.103246 38925041 PMC11255112

[B73] Zheng T. Gu Y. Wang J. Huang T. Gou L. Liu Y. (2025). Lactate-triggered histone lactylation contributes to podocyte epithelial-mesenchymal transition in diabetic nephropathy in mice. Chem. Biol. Interact. 408, 111418. 10.1016/j.cbi.2025.111418 39922521

[B74] Zhou Y. Zeng Y. Wang J. Zeng M. (2026). Lactate-lactylation axis as an emerging metabolic-epigenetic pathway in diabetic microvascular complications. Ageing Res. Rev. 118, 103100. 10.1016/j.arr.2026.103100 41831577

